# The Bacteroidetes Q-rule and glutaminyl cyclase activity increase the stability of extracytoplasmic proteins

**DOI:** 10.1128/mbio.00980-23

**Published:** 2023-09-26

**Authors:** Katarzyna Szczęśniak, Florian Veillard, Carsten Scavenius, Kamila Chudzik, Kinga Ferenc, Matthias Bochtler, Jan Potempa, Danuta Mizgalska

**Affiliations:** 1 Department of Microbiology, Faculty of Biochemistry, Biophysics and Biotechnology, Jagiellonian University, Kraków, Poland; 2 Department of Molecular Biology and Genetics, Aarhus University, Aarhus, Denmark; 3 International Institute of Molecular and Cell Biology, Warsaw, Poland; 4 Polish Academy of Sciences, Institute of Biochemistry and Biophysics, Warsaw, Poland; 5 Department of Oral Immunology and Infectious Diseases, University of Louisville School of Dentistry, Louisville, Kentucky, USA; Instituto Gulbenkian de Ciência, Oeiras, Portugal

**Keywords:** posttranslational modification, pyroglutamyl formation, proteolysis, *Porphyromonas gingivalis*, essential protein, periodontitis

## Abstract

In Gram-negative didermal species of the phylum Bacteroidetes, the majority of pre(pro)proteins exported across the cell membrane via the Sec system follow the Q-rule: a glutamine residue immediately downstream of a leader peptide is exposed as the new amino terminus by type I signal peptidases and converted to a pyroglutamate (also called oxyproline) residue by an inner membrane-associated glutaminyl cyclase (QC). Here, we show that the QC from *Porphyromonas gingivalis* is essential for growth in laboratory culture conditions. The lethal phenotype of QC deletion could not be rescued by an inactive variant of the enzyme, but it was rescued by QC orthologues from other species, despite their drastically lower activity toward a fluorescent reporter substrate. Replacement of glutamine after the signal peptide by an asparagine residue in selected QC substrates did not affect *P. gingivalis* viability but reduced the abundance of these proteins. Our data show that glutaminyl cyclization stabilizes *P. gingivalis* proteins, presumably protecting them from degradation by aminopeptidases. Loss of this protection is tolerated in individual substrates, but the complete loss in all Q-rule substrates is lethal, even in the absence of pressure from a host immune system.

**IMPORTANCE:**

Exclusively in the Bacteroidetes phylum, most proteins exported across the inner membrane via the Sec system and released into the periplasm by type I signal peptidase have N-terminal glutamine converted to pyroglutamate. The reaction is catalyzed by the periplasmic enzyme glutaminyl cyclase (QC), which is essential for the growth of *Porphyromonas gingivalis* and other periodontopathogens. Apparently, pyroglutamyl formation stabilizes extracytoplasmic proteins and/or protects them from proteolytic degradation in the periplasm. Given the role of *P. gingivalis* as the keystone pathogen in periodontitis, *P. gingivalis* QC is a promising target for the development of drugs to treat and/or prevent this highly prevalent chronic inflammatory disease leading to tooth loss and associated with severe systemic diseases.

## INTRODUCTION

Glutamine cyclization (pyroglutamyl formation) converts the N-terminal glutamine (Gln) residue of polypeptides into pyroglutamate (pGlu, 5-oxoproline), releasing ammonia as a side product. This posttranslational modification can occur spontaneously with some inorganic catalysts, but this route has little significance *in vivo* (1). More important is the reaction catalyzed by glutaminyl-peptide cyclotransferases, also known as glutaminyl cyclases (QCs). These enzymes are widespread in prokaryotes and eukaryotes and can be divided into two structurally unrelated classes (2 – 4). Type I (or plant type) QCs are found in plants, parasites, and most bacteria. They are represented by the papaya (*Carica papaya*) enzyme (CpQC) and feature a five-bladed β-propeller fold unrelated to peptidases (2, 5). In contrast, type II (animal type) QCs are found mainly in mammals but also in some bacteria. They are represented by the human (*Homo sapiens*) enzyme (HsQC) and are structurally related to aminopeptidases, featuring an α/β-hydrolase fold with an active site formed by aspartate (Asp) and glutamate (Glu) residues that together with a histidine (His) residue coordinate the Zn^2+^ ion (6).

Although QCs are found in most bacteria, the biological role of pyroglutamyl formation in bacteria is not well understood. The phylum Bacteroidetes is interesting in this context because most secreted proteins feature a Gln residue immediately downstream of the N-terminal signal peptide, which is cyclized in the periplasm once the signal peptide has been removed by signal peptidase I. Over the whole phylum, ~71% of signal peptidase I substrates follow this pattern (described as the Q-rule), but the proportion reaches 88% in some species. Proteomic analysis in *Porphyromonas gingivalis* and *Tannerella forsythia* confirmed the pyroglutamyl formation of several secreted proteins (4). Both species are recognized as important pathogens in the development and progression of periodontitis, a chronic inflammatory disease fueled by microbial dysbiosis on the tooth surface below the gum line (7).

In *P. gingivalis*, 77% of proteins with a type I signal peptide follow the Q-rule and are translocated across the inner membrane (IM) using the Sec translocon. They include structural and functional components of the type 9 secretion system (T9SS) and its cargo proteins, chaperones, proteins associated with the outer membrane (OM), and periplasmic proteins involved in peptide metabolism, peptidoglycan synthesis, and cell response regulation (Table 1). The N-terminal Gln of all these proteins (exposed after signal peptide cleavage) is cyclized by an animal-type QC (PG_2157, PG_RS09565) known as PgQC. Transposon mutagenesis has shown that *P. gingivalis* cannot survive and grow without the PgQC protein (8).

**TABLE 1:** The percentage of Q-positive hits in different functional categories of *P. gingivalis* proteins that are potential substrates of the type I signal peptidase*^f^*

Different functional categories	Total	N-terminal Q	% Q positive
T4SS	5	5	100.0
OMP-related functions*^a^*	73	64	87.7
T9SS^*c*^	43	37	86.0
Essential genes^*b*^	13	11^*e*^	84.6
Peptide catabolism^*d*^	19	16	84.2
Chaperones	6	5	83.3
All SPI substrates	210	162	77.1
Conserved hypothetical proteins	42	32	76.2
Biosynthesis peptidoglycan	9	6	66.7
Signaling and cell response and regulation	15	10	66.7
Carbohydrate catabolism	11	3	27.3
Biosynthesis LPS	4	1	25.0

^*a*^ Including all genes confirmed/predicted to be OMP and those necessary in their assembly.

^*b*^ Determined by Tn mutagenetic analysis (9).

^*c*^ That includes both secreted proteins and components of the T9SS apparatus including integral OM β-barrel proteins, periplasmic proteins, and cell surface-located proteins.

^*d*^ Including appropriate T9SS cargos.

^*e*^ Out of 11 genes deemed essential by Tn mutagenetic analysis, four *PG_0192*, *porG* (*PG_0189*), *motA* (*PG_0782*), and *PG_0937* were found recently as non-essential as their deletional mutants are viable (10 – 13).

^*f*^ The reference NZ_CP025932.1 *P. gingivalis* genomic sequence was used to extract all proteome data. The protein-coding sequences were screened using SignalP v6.0 to select only SPI substrates. Information was then gathered from different databases (PubMed, UniProt, KEGG and AlphaFold) as well as a BLAST homology search. Detailed information is presented in Table S5.

PgQC is a periplasmic lipoprotein anchored in the IM. We have previously tested its role in protein secretion and stability by mutating the N-terminal Gln to alanine (Ala) or asparagine (Asn) in several gingipains (Kgp, RgpA, and RgpB), a family of proteases secreted by *P. gingivalis* (4). Gingipains are expressed as inactive zymogens because their activity in the periplasm would be deleterious, so they mature into active proteases only after translocation to the bacterial surface (14). Nevertheless, mutants expressing the modified gingipains were viable, with only minor aberrations in the secretion and processing of RgpA and Kgp. This argues that the lethal phenotype of the PgQC deletion has a more complex underlying molecular background.

Here, we provide insight into the biological role of Gln cyclization in *P. gingivalis* by confirming that PgQC is an essential protein, investigating its mode of action, expressing a range of alternative QCs to test a spectrum of phenotypes relevant to *P. gingivalis* fitness, and analyzing the impact of blocking the pyroglutamyl formation of selected PgQC substrates with different cellular localizations and functions.

## MATERIALS AND METHODS

### Bacterial strains and general growth conditions

*Porphyromonas gingivalis* W83 cells were grown anaerobically in enriched trypticase soy broth (eTSB, 30 g/L) supplemented with yeast extract (5 g/L), l-cysteine (0.5 g/L), menadione (2 mg/L), and hemin (5 mg/L). We supplemented eTSB agar plates with 5% defibrinated sheep blood. *Escherichia coli* strain DH5α and *E. coli* S17-1 were grown in lysogeny broth (LB) (20 g/L). All bacterial strains used in this study are listed in Table S1.

### Genetic manipulation of *P. gingivalis*

We created *P. gingivalis* mutants by homologous recombination (15). For each mutant, a separate plasmid suitable for genomic integration was prepared in-house or synthesized commercially. For some experiments, the PgQC gene was expressed from vectors based on pTIO2-tet, which originates from pTIO-1 (16), using the *RagAB* promoter. Details of vector construction are provided in Supplemental Methods S1. The plasmids and primers used in this study are listed in Tables S2 and S3, respectively. All vectors were sequenced to confirm their integrity and introduced into *P. gingivalis* by electroporation or conjugation with *E. coli* S17-1. Transformants and transconjugants were propagated under antibiotic selection, and integration was confirmed by sequencing across the targeted genomic region.

The Asp126 residue in PgQC for D126A mutagenesis to generate a *P. gingivalis* strain expressing an inactive version of the enzyme was selected based on PgQC alignment with a Zn-dependent peptidase from *Bacteroides vulgatus* ATCC 8482 whose crystal structure at 1.8 A resolution (3GUX) is known. The motif AHWD^126^ containing His and Asp residues essential for peptidase activity is conserved in PgQC, and as expected, the recombinant enzyme PgQ^D126A^ expressed in *E. coli* had no activity on H-Gln-7-amino-4-methylcoumarin (H-Gln-AMC).

### Glutaminyl cyclase activity assay

Bacteria grown to the early stationary phase were harvested by centrifugation and washed once in cold phosphate-buffered saline (PBS). The cells were sonicated in a handheld UP200Ht homogenizer (Hielscher, Germany), and the protein concentration was determined using Pierce BCA Protein Assay Kit (Thermo Fisher Scientific, USA). QC activity was measured as previously described (4) using the fluorogenic substrate H-Gln-AMC (5 mM) pre-incubated with 10 µL of recombinant bacterial pyroglutamate aminopeptidase (12.5 U/mL, Unizyme Laboratories, Denmark) in 40 mM Tris-HCl and 400 mM KCl (pH 8.0) for 10 min at 30°C. The increase in fluorescence (λ_ex_ = 380 nm, λ_em_ = 460 nm) was recorded for 40 min at 30°C in a Flex Station 3 Multimode Microplate Reader (Molecular Devices, USA). The activity was normalized to the protein concentration in each sample.

### Subcellular fractionation of *P. gingivalis* cells

Stationary cell cultures were adjusted to OD_600_ = 1.4 with fresh eTSB containing 2 mM 2,2′-dithiothreitol (DTT; Sigma-Aldrich, USA) and were incubated for 15 min at room temperature to prepare the whole culture (WC) fraction. The cells were then centrifuged (5,000 *× g*, 20 min, 4°C), and the supernatant was clarified further (16,000 × *g*, 10 min, 4°C) to prepare the medium (M) fraction. The pellet was washed and resuspended in ice-cold PBS supplemented with 0.1 mM N_α_-tosyl-l-lysine chloromethyl ketone hydrochloride (TLCK; Sigma-Aldrich, USA) and cOmplete EDTA-free inhibitor mix (Roche, Switzerland). The cells were disrupted using a BT40/TS2/AA Constant System cell disruptor (Thermo Fisher Scientific, USA) at 30 kPa. The lysate was digested with 0.02 mg/mL DNase I (Roche) for 30 min to prepare the whole-cell extract (WCE) fraction. The lysate was centrifuged (150,000 × *g*, 1 h, 4°C) to separate the periplasmatic/cytoplasmatic fraction from the insoluble cell envelope including IM and OM, known as the cell membrane fraction. The cell envelope pellet was treated with 200 mM MgCl_2_ supplemented with 10% Triton X-100 for 30 min on ice to solubilize the IM and centrifuged as above. The collected supernatant was the IM fraction. The insoluble material was resuspended by sonication in PBS as the OM fraction. The obtained fractions were mixed with 0.1 mM TLCK, 1 mM DTT, 1 mM ethylenediaminetetraacetic acid (EDTA), and cOmplete EDTA-free inhibitor mix.

### Western blot analysis

Western blotting was performed on overnight whole bacteria cultures normalized according to OD_600_ measurements or on isolated cellular factions. Proteins were resolved by SDS-PAGE, and blotting was carried out using a Mini Trans-Blot device according to the manufacturer’s instructions (Bio-Rad Laboratories, USA). The blots were probed with anti-QC (4), anti-Strep (Novus Biologicals, USA), anti-His_6_ tag (GeneScript, USA), or in-house antibodies specific for PorQ, PorV, Kgp, RgpA, and RgpB. The secondary antibody was HRP-conjugated anti-rabbit or anti-mouse IgG, as appropriate (Sigma-Aldrich, USA). Signals were developed using Pierce ECL Western Blotting Substrate (Thermo Fisher Scientific, USA). The signals on photographed X-ray films (Agfa, Germany) were quantified by densitometry using Image Lab v6.1 (Bio-Rad Laboratories, USA).

### Gingipain activity assays

Bacteria cell cultures at the early stationary phase were collected, OD_600_ adjusted to 1, and gingipain amidolytic activity was determined in WC and cell-free medium (M) (after cells were removed by centrifugation) using N-benzoyl-DL-arginine p-nitroanilide (BApNA) and acetyl-Lys-pNA, respectively, for RgpA/B and Kgp. Briefly, samples were added to the assay buffer (200 mM Tris-HCl, 100 mM NaCl, 5 mM CaCl_2_, 10 mM L-cysteine, pH 7.6) in microplates to make a total volume of 190 µL and pre-incubated for 5 min at 37°C. Then, 10 µL of a substrate (final concentration, 1 mM) was added, and its enzymatic hydrolysis was recorded as the increase of OD_405_ per minute using Flex Station 3 Multimode Microplate Reader (Molecular Devices). Results are presented as mOD/min per 1 µL of a sample (mOD/min/µL).

### Quantitative RT-PCR analysis

Total RNA was isolated from bacteria in the late exponential phase (OD_600_ = 1.0) using the Total RNA Mini kit (A&A Biotechnology, Poland). Residual genomic DNA was removed using DNase I (A&A Biotechnology), and the RNA was purified again using the kit. We used 800 ng of re-purified RNA for cDNA synthesis with the High-Capacity cDNA Reverse Transcription Kit (Thermo Fisher Scientific). Quantitative RT-PCR was carried out in triplicate using SYBR Green JumpStart Taq ReadyMix (Sigma-Aldrich) and 333 nM of each primer (Table S3) on a CFX96 Touch Real-Time PCR Detection System (Bio-Rad Laboratories). Relative expression levels were calculated by normalizing each cycle threshold (Ct) value to the reference gene *rpoB* using the ΔΔCt method.

### Liquid chromatography tandem mass spectrometry

Membrane samples were analyzed by liquid chromatography-tandem mass spectrometry (LC-MS/MS) to find evidence of pGlu modifications. The samples were denatured, reduced, and alkylated in 20 mM Tris-HCl (pH 8) containing 8 M urea and 5 mM DTT, followed by the addition of 15 mM iodoacetamide. The reduced and alkylated samples were diluted and digested with trypsin at 37°C for 16 h. The tryptic peptides were micro-purified (17) before LC-MS/MS analysis on an eksigent nanoLC 415 system connected to a TripleTOF 6600 mass spectrometer (both from Sciex, Canada). The samples were trapped on a pre-column and then separated on a 15-cm analytical column, both packed with ReproSil-Pur C18-AQ 3 µm resin (Dr. Maisch, Germany). Peptides were eluted at a flow rate of 250 nL/min in a gradient of 5–35% solution B (0.1% formic acid in acetonitrile) lasting 30 min. Mass spectra were searched against *P. gingivalis* entries in UniProt or NCBI using an in-house Mascot search engine (Matrix Science, UK). Search parameters allowed semi-trypsin cleavage and carbamidomethylation as a fixed modification, with the peptide tolerance and MS/MS tolerance set to 10 ppm and 0.1 Da, respectively. Gln→pGlu (N-terminal Qln), Glu→pGlu (N-terminal Glu), and methionine oxidation were set as variable modifications.

### Protein purification and N-terminal sequencing

One liter of *P. gingivalis* strains PG_0449^his^ and PG_0449^Q22N,his^ was grown up to the early stationary phase in eTSB media at 37°C and in anaerobic conditions. Bacteria were harvested by centrifugation at 10,000 × *g* for 25 min, washed two times in PBS, and resuspended in PBS supplemented with Roche Proteases Inhibitors Cocktail and TLCK (10 µM). Bacteria were then lysed by sonication (3 cycles of 10  ×  5  s pulses at 17 W), and the obtained lysate was clarified by ultracentrifugation at 150,000 × *g* for 1  h. Soluble proteins were then dialyzed two times against 4 L of Ni-Sepharose binding buffer (20  mM sodium phosphate buffer pH 7.4, 500  mM NaCl, 20  mM imidazole, and 0.02% NaN_3_). Proteins of interest were purified by affinity chromatography on Ni-Sepharose High Performance matrix (GE Healthcare, Pittsburgh, PA, USA) and eluted in the same buffer supplemented with 500 mM imidazole. The protein concentration of the purified samples was determined by BCA Assay (Sigma-Aldrich), and its purity was determined by SDS-PAGE and Coomassie Blue staining.

For N-terminal sequencing, proteins separated by SDS-PAGE were electrotransferred onto a polyvinylidene difluoride membrane. Protein bands visualized by staining with Coomassie Brilliant Blue R-250 were excised and subjected to automated Edman degradation using a Procise 494 HT amino acid sequenator (Applied Biosystems, Carlsbad, CA, USA).

### Statistical analysis

Data were analyzed using Student’s *t*-test or one-way analysis of variance (ANOVA) with Bonferroni’s correction (comparison with control group) or Tukey’s correction (comparison across all groups) in GraphPad Prism v8 (GraphPad Software, USA). Statistical significance was assumed at *P* < 0.05.

## RESULTS

### QC deletion mutants are non-viable

**Fig 1:**
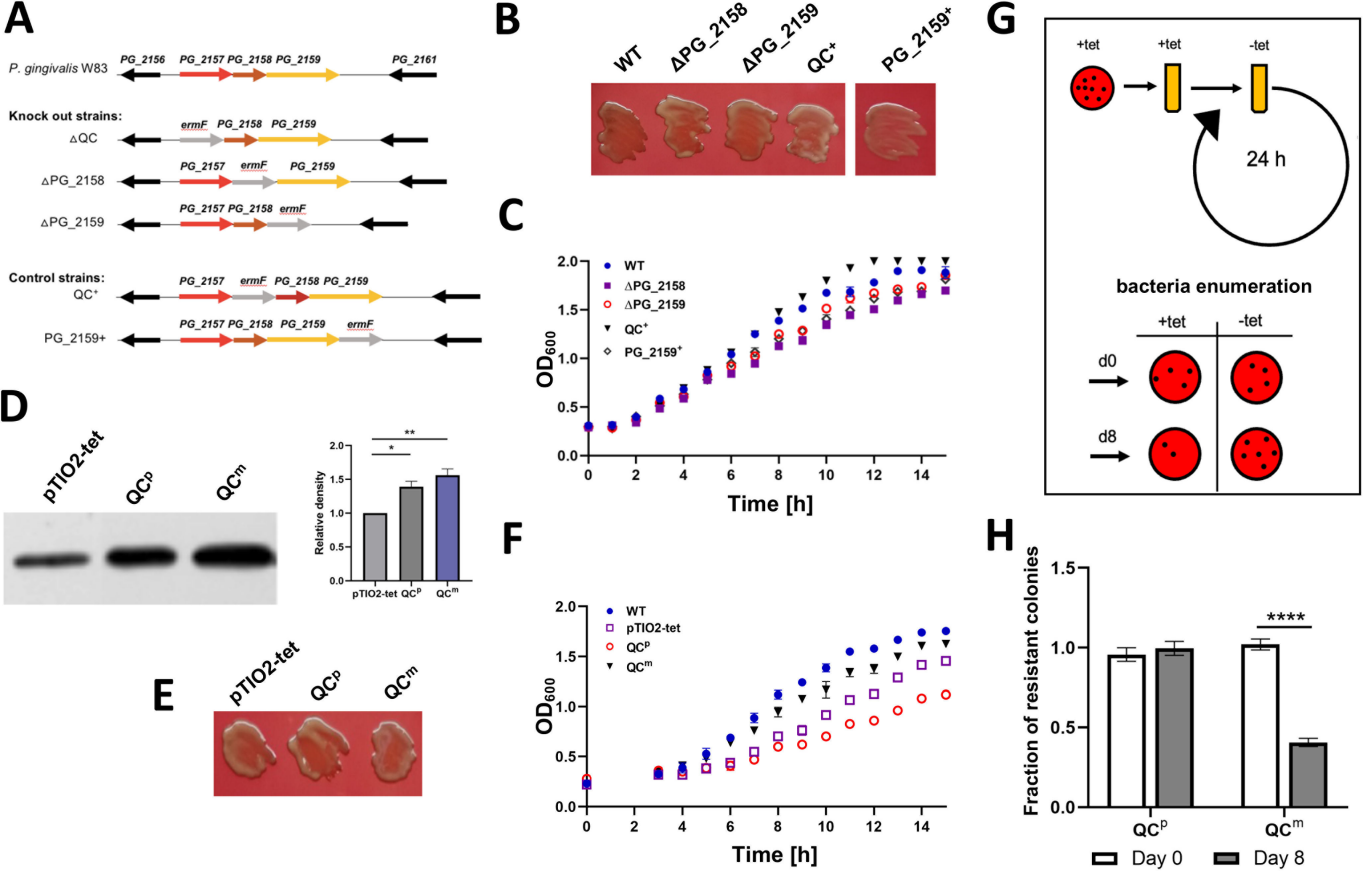
PgQC is an essential protein. (A) Arrangement of the three-gene operon in *P. gingivalis* and schematic description of control and mutant strains in this locus. Individual genes are shown in different colors: *PG_2157* encoding PgQC (red), *PG_2158* (burgundy), *PG_2159* encoding HemG (yellow), and *ermF*, the erythromycin cassette (gray). (B) Pigmentation phenotype of *P. gingivalis* wild-type (WT), ΔPG_2158, ΔPG_2159, QC^+^, and PG_2159^+^ strains plated on blood eTSB agar and photographed after anaerobic growth for 10 days. (C) Growth curve of the indicated *P. gingivalis* strains plotted by monitoring cell density (OD_600_) in liquid cultures for 16 h in triplicate. (D) Western blot (left panel) showing the abundance of PgQC (expected protein mass: 37.5 kDa) in QC^p^, QC^m^, and control strains (empty pTIO2-tet plasmid) in whole culture probed with anti-QC primary antibodies and HRP-conjugated anti-rabbit secondary antibodies (diluted 1: 20,000) and densitometry assessment of the intensity of immunoreactive bands (right panel) showing the mean from three independent experiments ± SEM. Statistical significance was calculated using one-way ANOVA (*P < 0.05, **P < 0.01). (E) Pigmentation phenotype of the *P. gingivalis* pTIO2-tet, QC^p^, and QC^m^ strains plated on blood eTSB plates with tetracycline (1 µg/mL) and photographed after anaerobic growth for 10 days. (F) Growth curve of the indicated *P. gingivalis* strains plotted by monitoring cell density (OD_600_) in liquid cultures for 16 h in triplicate. (G) Plasmid stability assay method. The QC^p^ and QC^m^ strains were cultured for eight generations in eTSB medium without antibiotics. On days 0 and 8, bacteria were plated at dilutions of 10^−6^, 10^−7^, and 10^−8^ on solidified medium with and without tetracycline. (H) Plasmid stability assay data presented as a proportion of cells that maintained tetracycline resistance. Data are means ± SEM (*n* = 3 technical replicates) and are representative of three independent experiments. Statistical significance was calculated by one-way ANOVA with *post hoc* Tukey’s correction (****P < 0.0001).

In agreement with transposon mutagenesis studies deeming PgQC essential (8), we were unable to create a viable PgQC deletion mutant (4). However, close inspection of the genomic locus revealed a complex arrangement of three open reading frames (*PG_2157*, *PG_2158*, and *PG_2159*), indicating that the PgQC gene (*PG_2157*) is the first of a three-gene operon (Fig. 1A). The accompanying genes are not well characterized but could be sensitive to polar effects when PgQC is mutated. *PG_2158* encodes a protein with an Fe-S center whose function is unknown, whereas *PG_2159* (*hemG*) encodes a protoporphyrin oxygenase involved in iron acquisition (18). To determine whether these genes are essential, we constructed two additional deletion mutants (Δ*PG_2158* and Δ*PG_2159*) and the control strains QC+ and PG_2159+ (Fig. 1A). In all these strains, the colony morphology and growth rate were indistinguishable from the wild-type strain, and gingipain activity was normal, with some increase for soluble Kgp activity (Fig. 1B and C; Fig. S1A and B). These results show that *PG_2158* and *PG_2159* are not essential for *P. gingivalis* at least *in vitro*, supporting our hypothesis that the pyroglutamyl formation of secreted proteins is essential for *P. gingivalis* growth.

### Plasmid stability assay confirms that QC is essential

To provide more evidence that PgQC is essential, we used a system in which gene significance can be inferred from the stability of a plasmid carrying a candidate essential gene in the absence of antibiotic selection pressure. For this purpose, we engineered a QC^m^ strain (m = merodiploid, reflecting the presence of two copies of the *PgQC* gene, one in the genome and the other on the plasmid). Next, we replaced the genomic *PgQC* coding sequence with the *ermF* cassette, resulting in a QC^p^ strain (p = plasmid, now the only copy of *PgQC*). Initial characterization of the QC^m^ and QC^p^ strains showed that the production of PgQC protein from the plasmid was enhanced compared to the control containing the empty plasmid (pTIO2-tet) (Fig. 1D). We did also observe some increase in gingipain activity (Fig. S1D and E), and as expected, there was no impairment in pigmentation phenotype (Fig. 1E), which is dependent on gingipain activity. The QC^p^ strain grew more slowly, and the generation time (*g*) was longer than the other strains (wild type, *g* = 2.42 h; pTIO2-tet, *g* = 3.19 h; QC^m^, *g* = 2.76 h; and QC^p^, *g* = 4.83 h). The stationary phase biomass of the QC^p^ strain was ~40% lower than that of the QC^m^ strain (Fig. 1F).

In the plasmid stability experiment, the QC^m^ and QC^p^ strains were maintained for eight generations in the absence of antibiotics. The number of cells carrying the expTIO_QC_tet plasmid was determined on days 0 and 8 by comparing the number of colonies on tetracycline vs control plates without antibiotics (Fig. 1G). We found that the QC^m^ strain was prone to curing, resulting in a lower number of antibiotic-resistant colonies on day 8 compared to day 0. In contrast, the QC^p^ strain maintained the plasmid even in the absence of selection, and the number of antibiotic-resistant colonies was similar on day 8 compared to day 0 (Fig. 1H). Taken together, these data confirm that PgQC expression is indispensable for *P. gingivalis* viability.

### PgQC is essential solely due to its enzymatic activity

**Fig 2:**
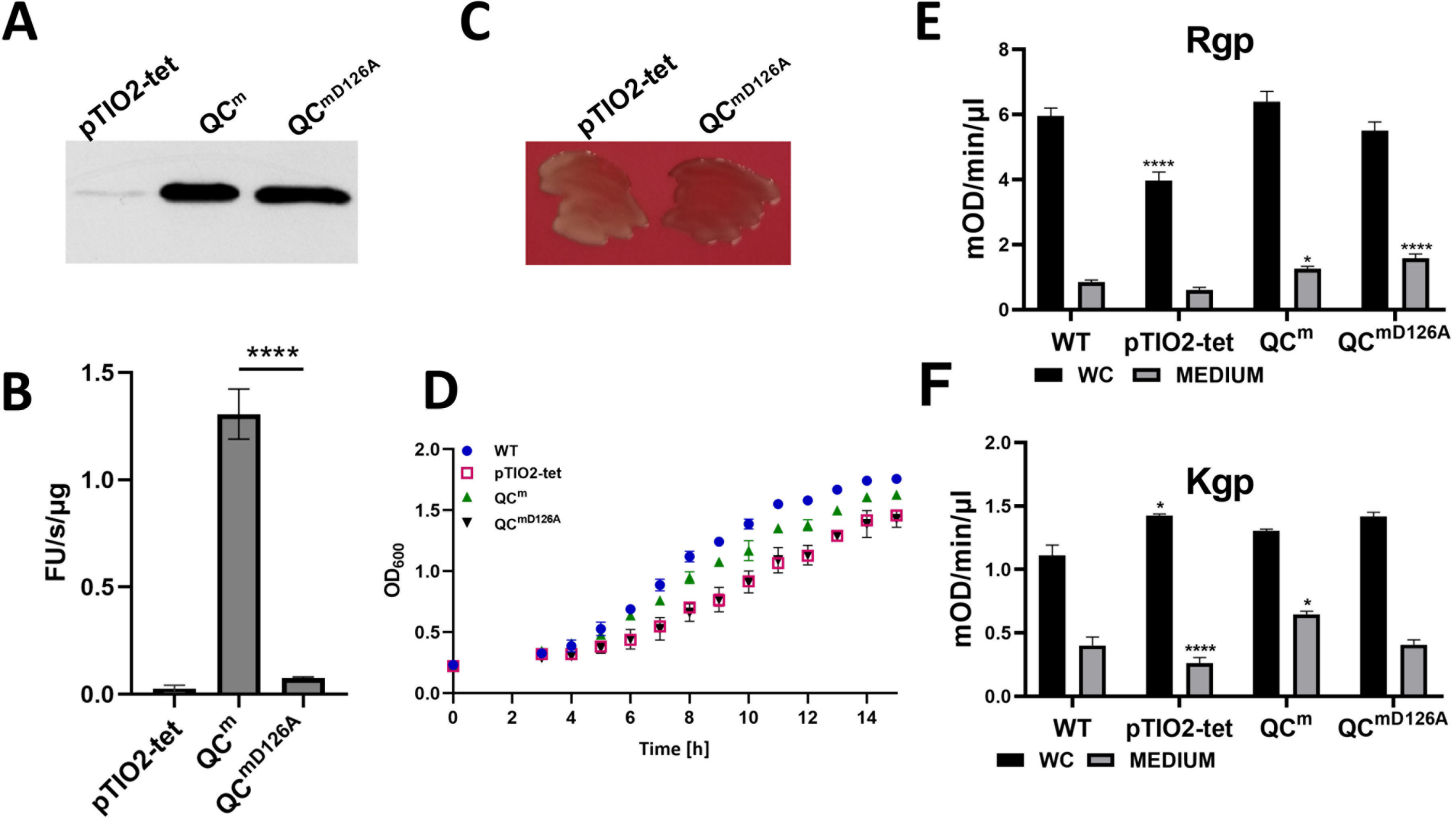
Importance of PgQC activity. (A) Representative western blot showing the abundance of PgQC in QC^m^, QC^mD126A^, and control strains (with empty pTIO2-tet plasmid) in whole culture probed with anti-QC primary antibodies and HRP-conjugated anti-rabbit secondary antibodies. (B) Comparison of QC activity in control (empty pTIO2-tet), QC^m^, and QC^mD126A^ strains measured with chromogenic substrate H-Gln-AMC (0.5 mM). Data are means ± SD (*n* = 3 technical replicates) and are representative of three independent experiments. Statistical significance was calculated using Student’s *t*-test (****P < 0.001). (C) Pigmentation phenotype of *P. gingivalis,* control (empty pTIO2-tet) and QC^mD126A^ strains plated on blood eTSB agar and photographed after anaerobic growth for 10 days. (D) Growth curve of the indicated *P. gingivalis* strains plotted by monitoring cell density (OD_600_) in liquid cultures for 16 h in triplicate. (E and F) Bacteria cell cultures at the early stationary phase were collected, OD_600_ adjusted to 1, and gingipain amidolytic activity was determined in whole culture (WC) and cell-free medium (M) using N-benzoyl-DL-arginine p-nitroanilide (BApNA) and acetyl-Lys-pNA, respectively, for RgpA/B (E) and Kgp (F). Data are means of three independent experiments ± SD. Statistical significance was calculated using Student’s *t*-test or one way ANOVA with Tukey’s correction (*P < 0.05, **P < 0.01, ***P < 0.001, ****P < 0.0001).

PgQC may be essential to *P. gingivalis* because its enzymatic activity is indispensable, or it may fulfill a non-catalytic role such as scaffolding or interacting with other proteins. Indeed, the spatial and functional coexistence of PgQC and the Sec translocon in the IM suggests that protein–protein interactions may contribute to the stabilization and/or function of Sec components (4). We therefore attempted to generate a *P. gingivalis* strain expressing catalytically inactive PgQC (mutation D126A). However, these attempts were unsuccessful, suggesting that PgQC enzymatic activity is necessary for *P. gingivalis* growth. This does not exclude the possibility that PgQC protein–protein interactions are also important, so we tested a possible dominant negative effect with an overexpression of the inactive enzyme (QC^D126A^) in wild-type *P. gingivalis* to see if it would inhibit growth. We introduced the plasmid overexpressing PgQC^D126A^ (expTIO-QC-tet-D126A) and found that the resulting QC^mD126A^ (mD126A = merodiploid overexpressing the inactive enzyme) mutant strain was fully viable. Like the QC^m^ strain, also, the QC^mD126^ strain produced much larger amounts of PgQC protein than the control strain with the empty plasmid (pTIO2-tet) (Fig. 2A), but, nevertheless, its enzymatic activity was similar to that of the control (Fig. 2B). Notably, the QC^mD126A^ mutant strain showed no difference in pigmentation phenotype (Fig. 2C), growth kinetics (Fig. 2D), or gingipain enzymatic activity (Fig. 2E and F) compared to the control strain. Moreover, proteomic analysis of pyroglutamyl formation revealed no significant differences between the wild-type and QC^mD126A^ strains (Table S4). Collectively, these results confirm that the importance of PgQC for *P. gingivalis* depends solely on the pyroglutamyl formation of proteins translocated across the IM by the Sec system.

Although these results suggest that physical interactions with Sec components are not an essential function of PgQC, its localization on the periplasmic side of the IM may be necessary for the modification of emerging N-terminal Gln residues as proteins are exported into the periplasm. Therefore, we replaced the Cys residue in the lipobox with Gln to produce mutant strain PgQC^C20Q^. This was phenotypically indistinguishable from the parental strain (Fig. S2A through C), and the QC activity was also at the same level (Fig. S2D). However, the protein was distributed between the IM and periplasm (Fig. S2E) in the form of full-length QC and a truncated version, probably with a cleaved signal peptide. This makes it impossible to determine whether the association between QC and the IM is important for its function.

### Heterologous expression of QC enzymes from other bacteroidetes species

**Fig 3:**
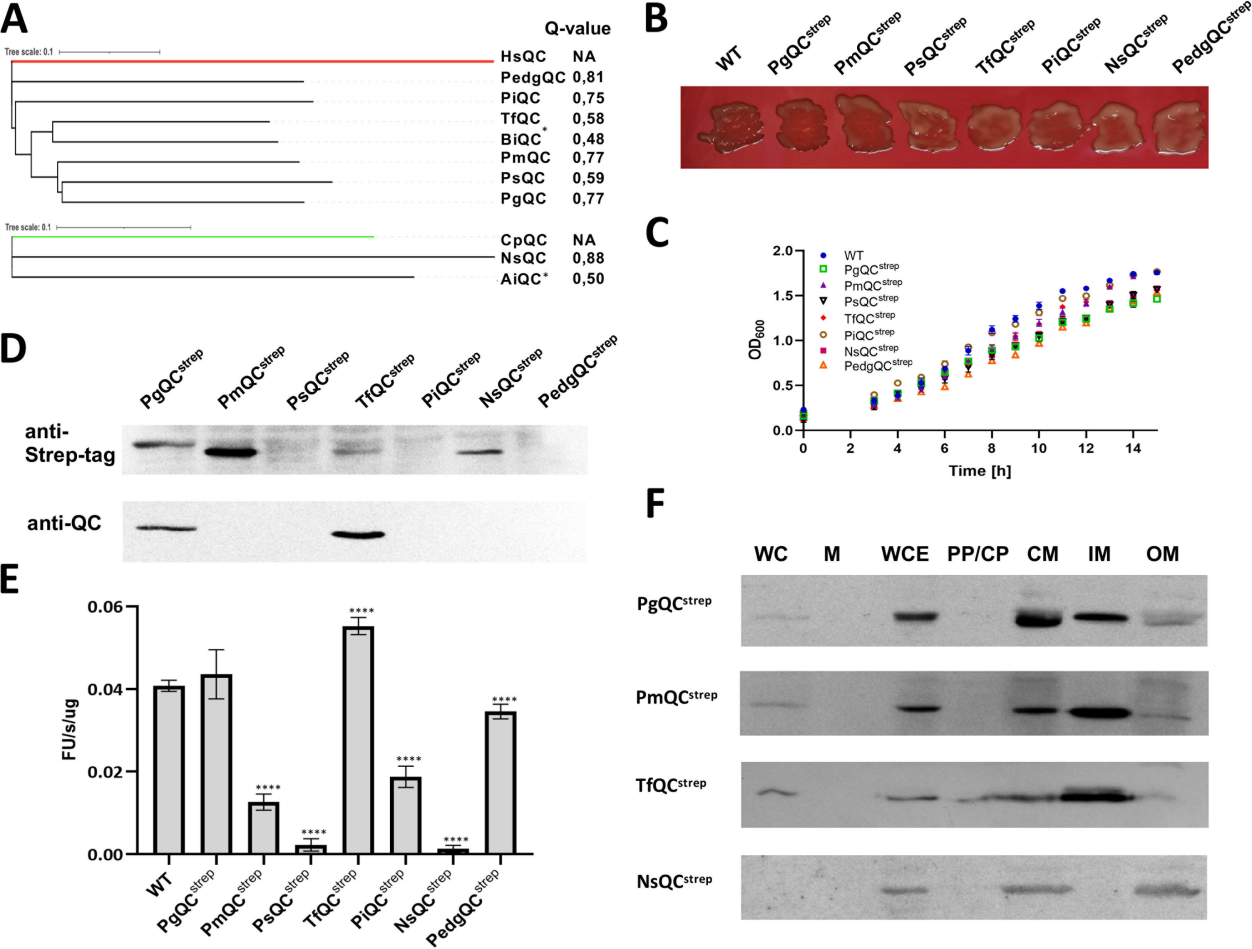
Heterologous expression of QC enzymes from other *Bacteroidetes* species. (A) Phylogenic trees showing the relationship between the heterologous QCs and the classical plant-type QC (from *Carica papaya*: CpQC, XP_021892140.1) and animal-type QC (from *Homo sapiens*: HsQC, AAH36721.1). The trees were assembled using Philo software on sequences aligned with M-Coffee and show calculated Q-values. (B) Pigmentation phenotype of *P. gingivalis* wild-type (WT) and PgQC^strep^ control strains together with strains expressing PmQC^strep^, PsQC^strep^, TfQC^strep^, PiQC^strep^, NsQC^strep^, and PedgQC^strep^. The strains were plated on blood eTSB agar and photographed after anaerobic growth for 10 days. (C) Growth curve of the indicated *P. gingivalis* strains plotted by monitoring cell density (OD_600_) in liquid cultures for 16 h in triplicate. (D) Western blots showing the abundance of QC protein in the whole culture of the control strain PgQC^strep^ (37.5 kDa) and strains expressing PmQC^strep^ (36.1 kDa), PsQC^strep^ (35.3 kDa), TfQC^strep^ (36.2 kDa), PiQC^strep^ (36 kDa), NsQC^strep^ (39.1 kDa), and PedgQC^strep^ (35.2 kDa). The blots were probed with anti-QC (diluted 1:20,000) and anti-Strep primary antibodies (1 µg/mL) and HRP-conjugated anti-rabbit secondary antibodies. (E) Comparison of QC activity in PgQC^strep^ and strains expressing PmQC^strep^, PsQC^strep^, TfQC^strep^, PiQC^strep^, NsQC^strep^, and PedgQC^strep^. Data are means ± SD (*n* = 3 technical replicates) and are representative of three independent experiments. Statistical significance was calculated by one-way ANOVA with *post hoc* Bonferroni correction (****P < 0.0001). (F) Representative western blots showing QC protein localization in strains PgQC^strep^, PmQC^strep^, TfQC^strep^, and NsQC^strep^. Fraction designations: WC, whole culture; M, medium; WCE, whole cell extract; PP/CP, periplasm/cytoplasm; CM, cell membranes; IM, inner membranes; OM, outer membranes. The enzymes were detected with an anti-Strep primary antibody with the exception of TfQC^strep^, which was detected with an anti-QC antibody.

Given the large proportion of *P. gingivalis* secreted proteins that follow the Q-rule, we investigated the essential function of PgQC in more detail by replacing it with orthologs from other Bacteroidetes species with different proportions of Q-rule proteins: *Porphyromonas macacae* (PmQC), *Porphyromonas somerae* (PsQC), *T. forsythia* (TfQC), *Prevotella intermedia* (PiQC), *Barnesiella intestinihominis* (BiQC), and *Pedobacter ginsenosidimutan* (PedgQC). In addition to these animal-type QCs, we also replaced PgQC with the plant-type QCs expressed by *Nonlabens sediminis* (NsQC) and *Alistipes indistinctus* (AiQC). This allowed us to test QCs with a high level of sequence and structural divergence (Fig. 3A; Fig. S3A). The mutant strains were constructed by in-phase substitution of the PgQC coding sequence downstream of the lipobox with the coding sequence of heterologous QCs (without the signal peptide, but including a C-terminal Strep-tag).

Most of the substitutions yielded viable *P. gingivalis* mutant strains with colony morphology, generation time, stationary phase biomass, and gingipain activity indistinguishable from the parental strain (Fig. 3B and C; Fig. S3B and C). However, mutant strains were not recovered for AiQC or BiQC. The presence of PsQC^strep^, PiQC^strep^, and PedgQC^strep^ could not be detected on western blots probed with anti-Strep or anti-QC antibodies (Fig. 3D), but all mutant strains produced measurable QC activity that differed significantly from strain to strain, with the lowest values in the strains expressing NsQC and PsQC (Fig. 3E). Remarkably, although the QC activity was 44-fold lower in NsQC and 22-fold lower in PsQC compared to wild-type *P. gingivalis*, this was still sufficient for the pyroglutamyl formation of Sec-exported proteins at a level that supported normal bacterial growth (Table S4). Although the QC activity of *P. gingivalis* mutant strains expressing TfQC and PiQC correspond well to the activity of native *T. forsythia* and *P. intermedia* strains (19), we cannot exclude the possibility that NsQC and PsQC expressed by *P. gingivalis* are fully active on physiological substrates but exert a minute activity against the reference synthetic substrate.

**Fig 4:**
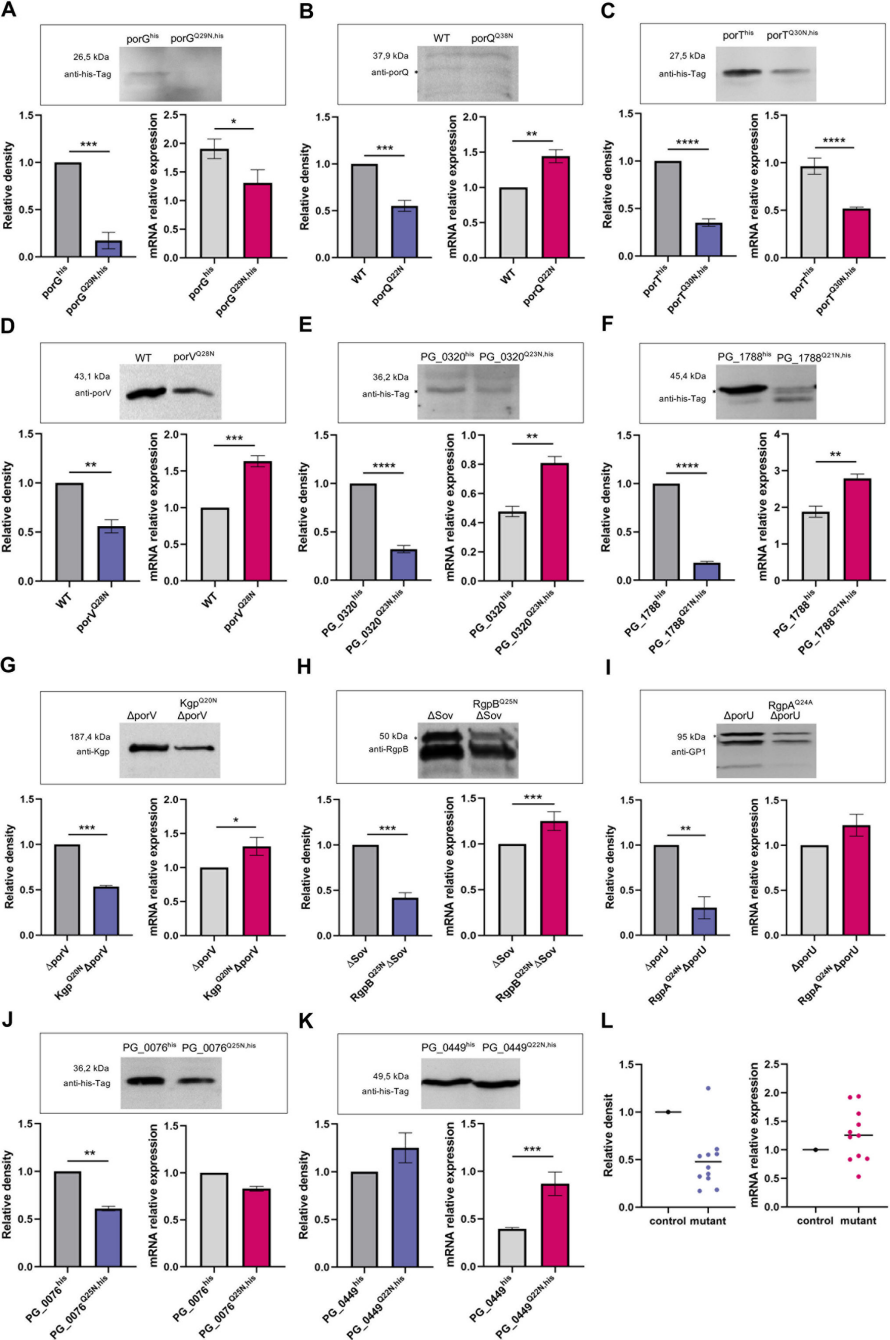
The significance of N-terminal pGlu for individual proteins. (A-K) Representative western blots of the whole culture extracts from different mutant *P. gingivalis* strains in which the N-terminal Gln cyclization of various proteins has been blocked. The proteins were detected with antibodies specific for the His_6_-tag (0.25 µg/mL) or in-house antibodies specific for PorQ (diluted 1:2,000), PorV (0.25 µg/mL), Kgp (diluted 1:2,000), RgpA, and RgpB (diluted 1:2,000). For secondary antibodies, we used HRP-conjugated anti-rabbit or anti-mouse antibodies as appropriate (diluted 1:20,000). The specific bands used for densitometry measurements are indicated with an asterisk. For each panel, the lower left (blue bar) shows western blot densitometry data, and the lower right (pink bar) shows the transcript abundance determined by semi-quantitative RT-PCR using RNA isolated during the mid-stationary phase and calculated using the ΔΔCt method normalized against the reference gene *rpoB*. In each case, data are means ± SEM (*n* = 3 technical replicates) and are representative of three independent experiments. Statistical significance was calculated using Student’s *t*-test or one-way ANOVA with Tukey’s correction (*P < 0.05, **P < 0.01, ***P < 0.001, ****P < 0.0001). (L) Normalized collected results from protein (left panel) and transcript (right panel) level analysis.

The subcellular location of heterologous QCs was determined by western blot, revealing that most were anchored into the IM, with only NsQC^strep^ found exclusively in the OM (Fig. 4F). Interestingly, the IM retention signal of *E. coli* lipoproteins is defined by Asp (+2 residue) following lipidated Cys (+1 residue), whereas glycine (Gly +2 or Gly +3) may fulfill the same function in *P. gingivalis* (20). Our experimental data provide supporting evidence because only NsQC was anchored into the OM and only this enzyme lacks a Gly residue in the Cys(+1)-Lys(+2)-Thr(+3) motif (Fig. S3A). It is remarkable that *P. gingivalis*, which is clearly dependent on PgQC, can survive despite the very low activity of heterologous enzymes, regardless of their type and subcellular location.

### The role of N-terminal pGlu formation

The unequivocally verified dependence of *P. gingivalis* on PgQC activity suggests that (i) there are one or more essential secreted proteins that require N-terminal modification for their stability or activity or (ii) the overall lack of pyroglutamyl formation may exert a cumulative detrimental effect. We, therefore, inspected all proteins with a signal peptide in the new reference genome NZ_CP025932.1. Where possible, we assigned a cellular localization, protein structure, molecular function, and biological process based on published research, databases (UniProt, KEGG, and AlphaFold), and BLAST homology searches. Among 210 secreted proteins, 164 (77.14%) appeared to follow the Q-rule and are, therefore, potential substrates of PgQC (Table 1; for details, see Table S5). These included 41 (20%) annotated as “conserved hypothetical proteins,” indicating that their function cannot be predicted because, at most, they are only distantly related to characterized proteins.

The very high prevalence of Gln following the signal peptide (Q-value) occurs among proteins predicted to be functionally and structurally associated with the OM (88.4%), T9SS components and cargo proteins (83.3%), remnants of the type 4 secretion system (T4SS) (100%), periplasmic chaperones (85.7%), and, surprisingly, the products of genes that were deemed essential by transposon mutagenesis (84.6%) (8). The Q-value of proteins involved in peptide catabolism, peptidoglycan biosynthesis, and cell response/regulation was 61.5–75%, but it was much lower for proteins involved in carbohydrate catabolism (27.3%) and lipopolysaccharide synthesis (25.0%). These potential PgQC substrates were found in all extracytoplasmic compartments. We also looked for conserved secondary structures at the N-terminus using Alpha-fold, but few crystallographic structures are available. The analysis revealed a tendency for β-sheet proteins, but this structure was predicted unambiguously only for a subgroup of OM β-barrel proteins.

We, therefore, tested the effect of replacing the key Gln with Ala or Asn in four of the OM β-barrel proteins (PorG, PorQ, PorT, and PorV) and two predicted periplasmic proteins (PG_0320 and PG_1788). Homologous *P. gingivalis* strains expressing each mutated protein were generated, and protein levels were determined by probing western blots with antibodies specific for the target proteins (PorQ and PorV) or antibodies specific for the C-terminal His_6_ tag (all other proteins). In all cases, the mutated protein accumulated to a significantly lower level than the normal protein in the control strain (Fig. 4A through K, left panels). This deficit was not due to transcriptional repression because, generally, the mutants showed elevated gene expression compared to the control (Fig. 4, right panels). The mutants were generally indistinguishable from the parental strains in terms of growth kinetics, pigmentation phenotype, and gingipain activity/distribution (Fig. S4 and S5). However, the *porT* ^Q30N,his^ strain was an exception, with slower growth than the control strains (Fig. S4C). Cumulatively, these results argue that pyroglutamyl formation is important for the stability of proteins exported to the periplasm and OM, apparently protecting them from proteolytic degradation and truncation, clearly visible in the case of PG_1788^Q21N,his^ (Fig. 4F). This was confirmed by the analysis of gingipains, which in the absence of a functional T9SS accumulate in the periplasm as multidomain single-chain proenzymes at a significantly lower level compared to control if the N-terminal Gln is replaced with Asn (Fig. 4G through I).

None of the mutant strains discussed above showed any loss of viability, but the target genes are known to be nonessential for *in vitro* growth on complex medium. We, therefore, repeated the same approach, but this time we mutated two genes (*PG_0076* and *PG_0449*) that were previously shown to be essential by transposon mutagenesis (8). In both mutant strains, the N-terminal Gln was replaced with Asn. Both mutant strains were viable and were indistinguishable from the parental strains in terms of growth, pigmentation, and gingipain activity (Fig. S4J, K, S5J and K). This was the case, despite the much lower level of PG_0076 protein and the truncation of PG_0449 (Fig. 4J and K).

In order to determine the impact of pyroglutamyl formation on extracytoplasmic proteins of *P. gingivalis*, we purified the modified PG_0449 protein from PG_0449^his^ and PG_0449^Q22N,his^ strains and subjected to N-terminal sequencing. The results revealed 13 amino acid truncations of the N-terminus of PG_0449 in the latter strain indicating N-terminal proteolytic degradation. Nevertheless, for other substrates, we cannot exclude a direct structure/function stabilizing effect of pyroglutame formation. Generally, the mutated genes were expressed at a higher level than their wild-type counterparts, but the increase in mRNA abundance was inversely correlated with the amount of protein (Fig. 4B, D through H and K). Although a lower rate of translation or a difference in mRNA stability cannot be excluded, this result strongly suggests the presence of a feedback loop that enhanced transcription but was unable to compensate for the degradation of the modified protein. This seems to be a common phenomenon when Gln is replaced, resulting in low levels of mutated proteins compared to the parental strains (Fig. 4L).

## DISCUSSION

### Aims

Bacteria from the phylum Bacteroidetes are unique among procaryotes in that cleavage of signal peptides by signal peptidase I exposes a glutamine residue as the new N-terminus in the majority of substrates. In prior work, we had already shown that this N-terminal glutamine residue is indiscriminately cyclized to a pyroglutamate residue (4). Saturating transposon mutagenesis data for *P. gingivalis* further indicated that the single glutaminyl cyclase in this species, a type II (animal type) QC, was likely to be essential (8). However, direct evidence for essentiality remained elusive, and—assuming the enzyme was essential—it remained unclear whether the enzyme activity was essential or whether the protein was required because of some other non-catalytic function. Finally, the biological role of glutaminyl cyclization remained unresolved. Our initial hypothesis that pyroglutamate formation was a sorting signal distinguishing between periplasmic and extracellular targeting could be ruled out by the observation that glutaminyl cyclization in *P. gingivalis* and other representative Bacteroidetes species were pervasive and occurred for proteins irrespective of their final destination (4).

### QC essentiality

In this work, we provide definitive experimental evidence that the QC from *P. gingivalis* is essential, and we show that the activity of the protein is required, even under laboratory growth conditions in rich medium. Furthermore, we could demonstrate that orthologues of the *P. gingivalis* QC could substitute for the endogenous QC, even though their activity, at least as assayed using a fluorogenic reporter substrate, was drastically lower than the activity of the endogenous protein. The observation that a seemingly residual QC activity was sufficient to rescue *P. gingivalis* growth in culture conditions was surprising because a lead QC inhibitor compound had previously been shown to block the growth of *P. gingivalis*, *T. forsythia*, and *P. intermedia* in laboratory cultures (21). The data may be reconciled with our data assuming that the lead compound reduced QC levels to below the 2% of activity measured in our assays for some of the QC orthologues. Alternatively, it is possible that the activity levels measured with our short fluorophore reporter peptide may not be representative of protein substrates of the cyclization pathway.

### Pyroglutamate formation to stabilize proteins

A biological role of glutamine cyclization was identified by our observation that glutaminyl cyclization appeared to stabilize selected Q-rule substrates. The protection of proteins by the pyroglutamyl formation of N-terminal Gln is illustrated here by the significantly less abundant mutant proteins in which the key Gln is replaced with Asn. These are 40%–80% less abundant than the normal proteins in wild-type *P. gingivalis*, even though the mutant genes show an increase in transcriptional activity (Fig. 4). It is likely that degradation would be even more profound if Gln was replaced with a hydrophobic residue to match the preference of the more potent peptidases DPP7 and DPP11, which cleave dipeptides from substrates with hydrophobic N-terminal residues (22). Remarkably, none of the strains expressing a Gln→Asn mutated protein, including those essential for *P. gingivalis*, showed a significant loss of vitality when growing *in vitro* in rich medium. This contrasts with our experimental confirmation that PgQC is essential for *P. gingivalis* growth. The discrepancy may reflect the residual amount of mutated Q-rule essential proteins as apparent in the case of PG_0078^Q25N^, which is sufficient for normal *P. gingivalis* growth. In addition, among eight essential proteins following the Q-rule (Table 1), we tested only two: PG_0076 and PG_0449. It is possible that one or more of the remaining proteins need N-terminal pGlu for its function or stability or that the cumulative effect of several protein depletions is necessary to evoke the detrimental effect. Alternatively, the lethal effect of PgQC inactivation may be due to the accumulation of partially degraded, inactive proteins in the periplasm.

### Pyroglutamate formation as a defense against aminopeptidases

On a technical level, our data only show that pyroglutamate formation stabilizes proteins, without providing a mechanism. It is extremely likely, however, that the protein stabilization is based on protection against aminopeptidases. The asaccharolytic nature of *P. gingivalis* metabolism makes this bacterium dependent on peptides generated in the extracellular milieu by an array of cell-surface endopeptidases (23). These peptides are translocated across the OM into the periplasm by a RagAB system (24) where they act as substrates for dipeptidyl-peptidases (DPP4, DPP5, DPP7, and DPP11) that release dipeptides from any N-terminus with a free α-amino group (9). DPPs work in concert with an acylpeptidyl-oligopeptidase and prolyl-tripeptidyl-peptidase to fragment oligopeptides (regardless of their sequence) into dipeptides (25). The latter are transported into the cytoplasm, where they are used as a carbon and energy source (26, 27). Interestingly, whereas DPP4 is widely distributed among prokaryotes, the Bacteroidetes genera *Tannerella*, *Prevotella*, *Porphyromonas, Capnocytophaga*, and *Bacteroides* also carry genes encoding homologs of DPP5, DPP7, and DPP11. The activity of these enzymes has been detected in *Porphyromonas endodontalis*, *T. forsythia*, and *P. intermedia* (26), the pathobionts contributing to the development of periodontitis. Notably, the growth of *T. forsythia* and *P. intermedia* as well as *P. gingivalis* was suppressed by a QC inhibitor (21), supporting our hypothesis that QC is an essential enzyme in species that follow the Q-rule. Signal peptidase I substrates in the phylum Bacteroidetes appear to have evolved to expose N-terminal Gln for pyroglutamyl formation by QC once the signal peptide is cleaved off (the Q-rule) in order to protect proteins exported into the periplasm or OM and secreted via the T9SS. Interestingly, the Q-rule has also been adopted by the recently discovered *P. gingivalis* phages to secrete proteins of yet unknown function (28). The intense proteolytic pressure in the periplasm and extracellular milieu of *P. gingivalis* and related Bacteroidetes species may well explain the applicability of the Q-rule to Bacteroidetes, but not other bacterial phyla, which may experience less pressure to stabilize periplasmic and extracellular proteins against proteolytic attack.

### Evolutionary conservation of pyroglutamate formation for protein stabilization

To our knowledge, the observation that protein cyclization serves as a protein stabilization mechanism is novel for bacteria. By contrast, it is a well-known phenomenon that, in eukaryotes, the cyclization of N-terminal residues often prolongs the half-life of proteins and peptides by protecting them from ubiquitous aminopeptidases (19, 29), as shown in studies focusing on chemoattractant proteins 1 and 3 (MCP-1 and MCP-3) and fractalkine (CX3CL1) (19, 30). However, this reaction also has broader biological significance. For example, malaria parasites evade the mosquito immune system by the pyroglutamyl formation of sporozoite surface proteins (31). In mammals, pyroglutamyl formation influences protein–protein interactions, as exemplified by ligands and receptor binding (32, 33). Importantly, extensive cyclization is related to pathological processes. It enhances the stability, hydrophobicity, and aggregation potential of the Aβ peptide underlying β-amyloid formation in Alzheimer’s disease (34); it forms pGlu79-α-synuclein, which promotes oligomerization and synucleinopathies in Parkinson’s disease (35, 36); and the modification of CD47 directly influences interactions with SIRPα to modulate the immunological surveillance mechanisms essential for the removal of cancer and senescent cells (37, 38). Together with the eukaryotic data, our data for bacteria make the case for deep evolutionary conservation of protein stabilization by glutaminyl cyclization.

## SUPPLEMENTAL MATERIAL

Method S1 and Tables S1 to S3 mbio.00980-23-s0001.docx Details of plasmids and strains generation, tables listing strains, plasmids and primers used in this study

Fig. S1 to Fig. S5 mbio.00980-23-s0002.docx Figures with additional strains phenotypic characterization, and protein sequence alignments.

Table S4 mbio.00980-23-s0003.xlsx Results from mass spectrometry.

Table S5 mbio.00980-23-s0004.xlsx All SPI substrate proteins from *P. gingivalis*.

ASM does not own the copyrights to Supplemental Material that may be linked to, or accessed through, an article. The authors have granted ASM a non-exclusive, world-wide license to publish the Supplemental Material files. Please contact the corresponding author directly for reuse.

## References

[1] Seifert F , Demuth H-U , Weichler T , Ludwig H-H , Tittmann K , Schilling S . 2015. Phosphate ions and glutaminyl cyclases catalyze the cyclization of glutaminyl residues by facilitating synchronized proton transfers. Bioorg Chem 60:98–101. doi:10.1016/j.bioorg.2015.04.005 25981125

[2] Schilling S , Wasternack C , Demuth H-U . 2008. Glutaminyl cyclases from animals and plants: a case of functionally convergent protein evolution. bchm 389:983–991. doi:10.1515/BC.2008.111 18979624

[3] Wu VW , Dana CM , Iavarone AT , Clark DS , Glass NL . 2017. Identification of glutaminyl cyclase genes involved in pyroglutamate modification of fungal lignocellulolytic enzymes. mBio 8:e02231-16. doi:10.1128/mBio.02231-16 28096492 PMC5241404

[4] Bochtler M , Mizgalska D , Veillard F , Nowak ML , Houston J , Veith P , Reynolds EC , Potempa J . 2018. The bacteroidetes Q-rule: pyroglutamate in signal peptidase I substrates. Front Microbiol 9:230. doi:10.3389/fmicb.2018.00230 29545777 PMC5837995

[5] Wintjens R , Belrhali H , Clantin B , Azarkan M , Bompard C , Baeyens-Volant D , Looze Y , Villeret V . 2006. Crystal structure of papaya glutaminyl cyclase, an archetype for plant and bacterial glutaminyl cyclases. J Mol Biol 357:457–470. doi:10.1016/j.jmb.2005.12.029 16438985

[6] Booth RE , Lovell SC , Misquitta SA , Bateman RC . 2004. Human glutaminyl cyclase and bacterial zinc aminopeptidase share a common fold and active site. BMC Biol 2:2. doi:10.1186/1741-7007-2-2 15028118 PMC368447

[7] Rajakaruna GA , Negi M , Uchida K , Sekine M , Furukawa A , Ito T , Kobayashi D , Suzuki Y , Akashi T , Umeda M , Meinzer W , Izumi Y , Eishi Y . 2018. Localization and density of Porphyromonas gingivalis and Tannerella forsythia in gingival and subgingival granulation tissues affected by chronic or aggressive periodontitis. Sci Rep 8:9507. doi:10.1038/s41598-018-27766-7 29934515 PMC6014976

[8] Hutcherson JA , Gogeneni H , Yoder-Himes D , Hendrickson EL , Hackett M , Whiteley M , Lamont RJ , Scott DA . 2016. Comparison of inherently essential genes of Porphyromonas gingivalis identified in two transposon-sequencing libraries. Mol Oral Microbiol 31:354–364. doi:10.1111/omi.12135 26358096 PMC4788587

[9] Shimoyama Y , Sasaki D , Ohara-Nemoto Y , Nemoto TK , Nakasato M , Sasaki M , Ishikawa T . 2023. Immunoelectron microscopic analysis of dipeptidyl-peptidases and dipeptide transporter involved in nutrient acquisition in Porphyromonas gingivalis *.* Curr Microbiol 80:106. doi:10.1007/s00284-023-03212-4 36797528

[10] Taguchi Y , Sato K , Yukitake H , Inoue T , Nakayama M , Naito M , Kondo Y , Kano K , Hoshino T , Nakayama K , Takashiba S , Ohara N . 2016. Involvement of an Skp-like protein PGN_0300, in the type IX secretion system of Porphyromonas gingivalis. Infect Immun 84:230–240. doi:10.1128/IAI.01308-15 26502912 PMC4693996

[11] Naito M , Tominaga T , Shoji M , Nakayama K . 2019. Pgn_0297 is an essential component of the type IX secretion system (T9SS) in Porphyromonas gingivalis: Tn-Seq analysis for exhaustive identification of T9SS-related genes. Microbiol Immunol 63:11–20. doi:10.1111/1348-0421.12665 30599082 PMC6590471

[12] Ho M-H , Lamont RJ , Xie H . 2017. Identification of streptococcus cristatus peptides that repress expression of virulence genes in Porphyromonas gingivalis. Sci Rep 7:1413. doi:10.1038/s41598-017-01551-4 28469253 PMC5431200

[13] Veith PD , Shoji M , Scott NE , Reynolds EC . 2022. Characterization of the O-Glycoproteome of Porphyromonas gingivalis *.* Microbiol Spectr 10:e0150221. doi:10.1128/spectrum.01502-21 34985300 PMC8729774

[14] Potempa J , Nguyen K-A . 2007. Purification and characterization of gingipains. CP Protein Science 49:21. doi:10.1002/0471140864.ps2120s49 18429320

[15] Bélanger M , Rodrigues P , Progulske-Fox A . 2007. Genetic manipulation of Porphyromonas gingivalis. Curr Protoc Microbiol Chapter 13:Unit13C.2. doi:10.1002/9780471729259.mc13c02s05 18770611

[16] Tagawa J , Inoue T , Naito M , Sato K , Kuwahara T , Nakayama M , Nakayama K , Yamashiro T , Ohara N . 2014. Development of a novel plasmid vector pTIO-1 adapted for electrotransformation of Porphyromonas gingivalis. J Microbiol Methods 105:174–179. doi:10.1016/j.mimet.2014.07.032 25102110

[17] Rappsilber J , Mann M , Ishihama Y . 2007. Protocol for micro-purification, enrichment, pre-fractionation and storage of peptides for proteomics using StageTips. Nat Protoc 2:1896–1906. doi:10.1038/nprot.2007.261 17703201

[18] Kusaba A , Ansai T , Akifusa S , Nakahigashi K , Taketani S , Inokuchi H , Takehara T . 2002. Cloning and expression of a Porphyromonas gingivalis gene for protoporphyrinogen oxidase by complementation of a hemG mutant of Escherichia coli. Oral Microbiol Immunol 17:290–295. doi:10.1034/j.1399-302x.2002.170505.x 12354210

[19] Cynis H , Hoffmann T , Friedrich D , Kehlen A , Gans K , Kleinschmidt M , Rahfeld J-U , Wolf R , Wermann M , Stephan A , Haegele M , Sedlmeier R , Graubner S , Jagla W , Müller A , Eichentopf R , Heiser U , Seifert F , Quax PHA , de Vries MR , Hesse I , Trautwein D , Wollert U , Berg S , Freyse E-J , Schilling S , Demuth H-U . 2011. The isoenzyme of glutaminyl cyclase is an important regulator of monocyte infiltration under inflammatory conditions. EMBO Mol Med 3:545–558. doi:10.1002/emmm.201100158 21774078 PMC3377097

[20] Gabarrini G , Grasso S , van Winkelhoff AJ , van Dijl JM . 2020. Gingimaps: protein localization in the oral pathogen Porphyromonas gingivalis Microbiol Mol Biol Rev 84:e00032-19. doi:10.1128/MMBR.00032-19 31896547 PMC6941882

[21] Taudte N , Linnert M , Rahfeld J-U , Piechotta A , Ramsbeck D , Buchholz M , Kolenko P , Parthier C , Houston JA , Veillard F , Eick S , Potempa J , Schilling S , Demuth H-U , Stubbs MT . 2021. Mammalian-like type II glutaminyl cyclases in Porphyromonas gingivalis and other oral pathogenic bacteria as targets for treatment of periodontitis. J Biol Chem 296:100263. doi:10.1016/j.jbc.2021.100263 33837744 PMC7948796

[22] Rouf SMA , Ohara-Nemoto Y , Hoshino T , Fujiwara T , Ono T , Nemoto TK . 2013. Discrimination based on Gly and Arg/Ser at position 673 between Dipeptidyl-Peptidase (DPP) 7 And DPP11, widely distributed DPPs in pathogenic and environmental gram-negative bacteria. Biochimie 95:824–832. doi:10.1016/j.biochi.2012.11.019 23246913

[23] Potempa J , Sroka A , Imamura T , Travis J . 2003. Gingipains, the major cysteine proteinases and virulence factors of Porphyromonas gingivalis: structure, function and assembly of multidomain protein complexes. Curr Protein Pept Sci 4:397–407. doi:10.2174/1389203033487036 14683426

[24] Madej M , White JBR , Nowakowska Z , Rawson S , Scavenius C , Enghild JJ , Bereta GP , Pothula K , Kleinekathoefer U , Baslé A , Ranson NA , Potempa J , van den Berg B . 2020. Structural and functional insights into oligopeptide acquisition by the RagAB transporter from Porphyromonas gingivalis. Nat Microbiol 5:1016–1025. doi:10.1038/s41564-020-0716-y 32393857 PMC7610489

[25] Ohara-Nemoto Y , Shimoyama Y , Ono T , Sarwar MT , Nakasato M , Sasaki M , Nemoto TK . 2022. Expanded substrate specificity supported by P1′ and P2′ residues enables bacterial dipeptidyl-peptidase 7 to degrade bioactive peptides. J Biol Chem 298:101585. doi:10.1016/j.jbc.2022.101585 35032549 PMC8851246

[26] Ohara-Nemoto Y , Sarwar MT , Shimoyama Y , Kobayakawa T , Nemoto TK . 2020. Preferential dipeptide incorporation of Porphyromonas gingivalis mediated by proton-dependent oligopeptide transporter (Pot). FEMS Microbiol Lett 367:fnaa204. doi:10.1093/femsle/fnaa204 33338236

[27] Nemoto TK , Ohara Nemoto Y . 2021. Dipeptidyl‐peptidases: key enzymes producing entry forms of extracellular proteins in asaccharolytic periodontopathic bacterium Porphyromonas gingivalis. Mol Oral Microbiol 36:145–156. doi:10.1111/omi.12317 33006264 PMC8048996

[28] Matrishin CB , Haase EM , Dewhirst FE , Mark Welch JL , Miranda-Sanchez F , Chen T , MacFarland DC , Kauffman KM . 2023. Phages are unrecognized players in the ecology of the oral pathogen Porphyromonas gingivalis. Microbiome 11:161. doi:10.1186/s40168-023-01607-w 37491415 PMC10367356

[29] Morty RE , Bulau P , Pellé R , Wilk S , Abe K . 2006. Pyroglutamyl peptidase type I from trypanosoma brucei: a new virulence factor from African trypanosomes that de-blocks regulatory peptides in the plasma of infected hosts. Biochem J 394:635–645. doi:10.1042/BJ20051593 16248854 PMC1383713

[30] Kehlen A , Haegele M , Böhme L , Cynis H , Hoffmann T , Demuth H-U . 2017. N-terminal pyroglutamate formation in CX3CL1 is essential for its full biologic activity. Biosci Rep 37:BSR20170712. doi:10.1042/BSR20170712 28739588 PMC5634468

[31] Franke-Fayard B , Marin-Mogollon C , Geurten FJA , Chevalley-Maurel S , Ramesar J , Kroeze H , Baalbergen E , Wessels E , Baron L , Soulard V , Martinson T , Aleshnick M , Huijs ATG , Subudhi AK , Miyazaki Y , Othman AS , Kolli SK , Lamers OAC , Roques M , Stanway RR , Murphy SC , Foquet L , Moita D , Mendes AM , Prudêncio M , Dechering KJ , Heussler VT , Pain A , Wilder BK , Roestenberg M , Janse CJ . 2022. Creation and preclinical evaluation of genetically attenuated malaria parasites arresting growth late in the liver. NPJ Vaccines 7:139. doi:10.1038/s41541-022-00558-x 36333336 PMC9636417

[32] Schilling S , Niestroj AJ , Rahfeld J-U , Hoffmann T , Wermann M , Zunkel K , Wasternack C , Demuth H-U . 2003. Identification of human glutaminyl cyclase as a metalloenzyme. J Biol Chem 278:49773–49779. doi:10.1074/jbc.M309077200 14522962

[33] Wu Z , Weng L , Zhang T , Tian H , Fang L , Teng H , Zhang W , Gao J , Hao Y , Li Y , Zhou H , Wang P . 2019. Identification of glutaminyl cyclase isoenzyme isoQC as a regulator of SIRPα-CD47 axis. Cell Res 29:502–505. doi:10.1038/s41422-019-0177-0 31089204 PMC6796872

[34] Cynis H , Scheel E , Saido TC , Schilling S , Demuth H-U . 2008. Amyloidogenic processing of amyloid precursor protein: evidence of a pivotal role of glutaminyl cyclase in generation of pyroglutamate-modified amyloid-beta. Biochemistry 47:7405–7413. doi:10.1021/bi800250p 18570439

[35] Hartlage-Rübsamen M , Bluhm A , Moceri S , Machner L , Köppen J , Schenk M , Hilbrich I , Holzer M , Weidenfeller M , Richter F , Coras R , Serrano GE , Beach TG , Schilling S , von Hörsten S , Xiang W , Schulze A , Roßner S . 2021. A glutaminyl cyclase-catalyzed α-Synuclein modification identified in human synucleinopathies. Acta Neuropathol 142:399–421. doi:10.1007/s00401-021-02349-5 34309760 PMC8357657

[36] Bluhm A , Schrempel S , Moceri S , Stieler J , Feja M , Schilling S , Schulze A , von Hörsten S , Hartlage-Rübsamen M , Richter F , Roßner S . 2022. Alpha synuclein processing by MMP-3 – implications for synucleinopathies. Behav Brain Res 434:114020. doi:10.1016/j.bbr.2022.114020 35870616

[37] Logtenberg MEW , Jansen JHM , Raaben M , Toebes M , Franke K , Brandsma AM , Matlung HL , Fauster A , Gomez-Eerland R , Bakker NAM , van der Schot S , Marijt KA , Verdoes M , Haanen JBAG , van den Berg JH , Neefjes J , van den Berg TK , Brummelkamp TR , Leusen JHW , Scheeren FA , Schumacher TN . 2019. Glutaminyl cyclase is an enzymatic modifier of the CD47- SIRPα axis and a target for cancer Immunotherapy. Nat Med 25:612–619. doi:10.1038/s41591-019-0356-z 30833751 PMC7025889

[38] Schloesser D , Lindenthal L , Sauer J , Chung K-J , Chavakis T , Griesser E , Baskaran P , Maier-Habelsberger U , Fundel-Clemens K , Schlotthauer I , Watson CK , Swee LK , Igney F , Park JE , Huber-Lang MS , Thomas M-J , El Kasmi KC , Murray PJ . 2023. Senescent cells suppress macrophage-mediated corpse removal via upregulation of the CD47-QPCT/L axis. J Cell Biol 222:e202207097. doi:10.1083/jcb.202207097 36459066 PMC9723804

