# Supplementary material for: The Bacteroidetes Q-rule and glutaminyl cyclase activity increase the stability of extracytoplasmic proteins
Source: mBio. 2023 Sep 26;14(5):e00980-23. doi: 10.1128/mbio.00980-23 (PMC10653852; doi:10.1128/mbio.00980-23)
Supplement: Method S1 and Tables S1 to S3 — Details of plasmids and strains generation, tables listing strains, plasmids and primers used in this study [file mbio.00980-23-s0001.docx]

**SUPPLEMENTARY INFORMATION**

***Table of Contents 1***

1. ***Supplementary methods***

***Method S1. Details for plasmid construction and genomic mutagenesis 2***

***2. Supplementary tables 8***

***Supplementary Table S1 8***

***Supplementary Table S2 9***

***Supplementary Table S3 11***

***4. Supplementary references 14***

***­­­­­­–––––––––––––––––––––––––––––––––––––––––––––––––––––––––––––––––––––––––––***

1. ***Supplementary methods.***

***Method S1. Plasmid construction for* P. gingivalis *genomic mutagenesis.***

Suicide vectors for genetic manipulation were manufactured in-house based on the pUC19 plasmid backbone with three PCR amplified inserts, in the form of antibiotic cassettes flanked by two sequences amplified from *P. gingivalis* W83 genomic DNA (with Phusion polymerase, Thermo Scientific, USA). The tetracycline (*tetQ*) or erythromycin (*ermF*) resistance genes were amplified from plasmids pT-COW (1) and pURgpB-E, respectively (2). For some vectors, where erythromycin was sandwiched between operon-forming genes, the *ermF* gene was cloned without any regulatory sequences (only its open reading frame (ORF) was amplified). Vectors were constructed either using the classical restriction enzymes-based cloning and T4 ligase approach (all from Thermo Scientific, USA) or by the homology-based Gibson assembly method (In-Fusion® HD Cloning Kit (Takara Bio Europe, Japan). If necessary, plasmids were further genetically modified using PCR-based SLIM mutagenesis (3) or Gibson assembly (4). For heterologous QC substitution experiments, selected QC genes from various species were synthesized commercially with codon usage optimized for expression in *P. gingivalis* or amplified from adequate genomic DNA. Finally, all vectors constructed were sequenced and transformed into electrocompetent *P. gingivalis* cells. After 24 h anaerobic incubation in eTSB, cells were plated on appropriate selective blood agar plates supplemented with either tetracycline or erythromycin. Obtained colonies were propagated and analyzed for proper integration by targeted region sequencing. Details for plasmid construction used for genome modifications:

- **pQCdel-E**, no strain obtained - upstream fragment (UP) (1067 bp): QC_AF_EcoRI, QC_AR_KpnI; downstream fragment (DW) (947 bp): QC_BF_XbaI, QC_BR_HindIII; antibiotic: ORF of erythromycin (802 bp): Erm_F_KpnI, Erm_R_XbaI.

- **pQCdelKX-E**, no strain obtained – derivative of pQCdel-E plasmid, devoted of two restriction sites KpnI (localized between UP and *ermF* ORF) and XbaI (localized between *ermF* ORF and DW) by the SLIM-based method (3).

- **pUCQC2** for strain **QC^+^**- UP (1067 bp): QCc1_A-F and QCc1_A-R; DW (947 bp): QCc2_B-F and QCc2_E-R; antibiotic: ORF of erythromycin (802 bp): QCc1_E-F and QCc2_E-R.

- **pUCQC1** for strain **ΔPG_2158**- UP (1067 bp): QCc1_A-F and QCc1_A-R; DW (1407 bp): QCc1_B-F and QCc1_B-R; antibiotic: ORF of erythromycin (802 bp): QCc1_E-F and QCc1_E-R.

- **pUCQC3** for strain **ΔPG_2159**- UP (1067 bp): QCc1_A-F and QCc3_A-R; DW (885 bp): QCc3_B-F and QCc3_B-R; antibiotic: ORF of erythromycin (802 bp): QCc3_E-F and QCc3_E-R.

- **pUCQC5**, strain **ΔPG_2159**- UP (942 bp): QCc5_A-F and QCc5_A-R; DW (872 bp): nQCc3_B-F and QCc3_B-R; antibiotic: erythromycin gene: (1184 bp): QCc5_E-F and QCc3_E-R.

- **pUCQC2-D126A**, no strain obtained - mutation of the PgQC catalytic residue (Asp126 Ala) by the SLIM-based method with primers QC- D126A.

- **pUCQC2-Strep** for strain **PgQC^strep^** - based on the pUCQC2 plasmid, the Strep-tag insertion was obtained by Gibson assembly of PCR amplified pUCQC2 using QCstreptagR and QCstreptagF primers.

- **pUCQCdelKX-E_pmac-strep** for strain **PmQC^strep^** - based on pQCdelKX-E, substitutes PgQC with the *Porphyromonas macacae* QC gene (PmQC) in the *P. gingivalis* genome. pQCdelKX-E was linearized with primers QC_pUC_Fr and QCdelA-R and PmQC was amplified from a commercially synthesized gene (based on WP_052080475.1, *P. gingivalis* optimized codon usage, SP deprived) with GQC.sp_Fr and QC_pmac_Rv. Next, the C-terminal Strep-tag was inserted with Gibson assembly of QC_strepG_Fr with a pmac_strepG_Rv amplified intermediate vector.

- **pUCQCdelKX-E_psom-strep** for strain **PsQC^strep^** - based on pQCdelKX-E, substitutes PgQC with the *Porphyromonas somerae* QC gene (PsQC) in the *P. gingivalis* genome. pQCdelKX-E was linearized with primers QC_pUC_Fr and QCdelA-R and PsQC was amplified from a commercially synthesized gene (based on KXB76915.1, *P. gingivalis* optimized codon usage, SP deprived) with GQC.sp_Fr and QC_psom_Rv. Next, the C-terminal Strep-tag was inserted with Gibson assembly of the QC_strepG_Fr with a psom_strepG_Rv amplified intermediate vector.

- **pUCQCdelKX-E_pedg-strep** for strain **PedgQC^strep^** - based on pQCdelKX-E, substitutes PgQC with the *Pedobacter ginsenosidimutan* QC gene (PedgQC) in the *P. gingivalis* genome. pQCdelKX-E was linearized with primers QC_pUC_Fr and QCdelA-R and PedgQC gene was amplified from a commercially synthesized gene (based on WP_057931203.1, *P. gingivalis* optimized codon usage, SP deprived) with GQC.sp_Fr and QC_pedg_Rv. Next, the C-terminal Strep-tag was inserted with Gibson assembly of the QC_strepG_Fr and pedg_strepG_Rv amplified intermediate vector.

- **pUCQCdelKX-E_nsed-strep** for strain **NsQC^strep^** - based on pQCdelKX-E, substitutes PgQC with the *Nonlabens sediminis* QC gene (NsQC) in the *P. gingivalis* genome. pQCdelKX-E was linearized with primers QC_pUC_Fr and QCdelA-R and the NsQC gene was amplified from a commercially synthesized gene (based on WP_042277877.1, *P. gingivalis* optimized codon usage, SP deprived) with GQC.sp_Fr and QC_nsed_Rv. Next, C-terminal Strep-tag was inserted with Gibson assembly of the QC_strepG_Fr and nsed_strepG_Rv amplified intermediate vector.

- **pUCQCdelKX-E_aind-strep**, no strain obtained - based on pQCdelKX-E, substitutes the PgQC with *Alistipes indistinctus* QC gene (AiQC) in the *P. gingivalis* genome. pQCdelKX-E was linearized with primers QC_pUC_Fr and QCdelA-R and the AiQC gene was amplified from a commercially synthesized gene (based on WP_118061326.1, *P. gingivalis* optimized codon usage, without SP) with GQC.sp_Fr and QC_aind_Rv. Next, C-terminal Strep-tag was inserted with Gibson assembly of the QC_strepG_Fr and aind_strepG_Rv amplified intermediate vector.

- **pUCQCdelKX-E_bint-strep**, no strain obtained - based on pQCdelKX-E, substitutes PgQC with the *Barnesiella intestinihominis* QC gene (BiQC) in the *P. gingivalis* genome. pQCdelKX-E was linearized with primers QC_pUC_Fr and QCdelA-R and the BiQC gene was amplified from a commercially synthesized gene (based on WP_008861433.1, *P. gingivalis* optimized codon usage, without SP) with GQC.sp_Fr and QC_bint_Rv. Next, C-terminal Strep-tag was inserted with Gibson assembly of the QC_strepG_Fr and bint_strepG_Rv amplified intermediate vector.

**- pUCQCdelKX-E_tfor-strep** for strain **TfQC^strep^** - based on pQCdelKX-E_psom-strep, substitutes PgQC with the *Tannerella forsythia* QC gene (TfQC) in the *P. gingivalis* genome. pQCdelKX-E_psom-strep was linearized with primers QC_strepG_Fr and QC_PS_Rv and TforQC was amplified from *T. forsythia* ATCC 43037 genomic DNA (KKY61917.1, SP deprived) with QC_TF_Fr and QC_TF_Rv primers.

**- pUCQCdelKX-E_pint-strep** for strain **PiQC^strep^** - based on pQCdelKX-E_psom-strep, substitutes PgQC with the *Prevotella intermedia* QC gene (PiQC) in the *P. gingivalis* genome. pQCdelKX-E_psom-strep was linearized with primers QC_strepG_Fr and QC_PS_Rv while PintQC was amplified from *P. intermedia* 17 genomic DNA (AFJ09628.1, without SP) using QC_PI_Fr and QC_PI_Rv primers.

- **pg22-E_pg22-Q28N**, strain **pg22-E_pg23-Q28N** *-* based on plasmid pg22-E (2) that introduces the erythromycin cassette directly preceding the *porV* gene. Gln28 to Asn (Q28N) was mutated with primers ***pg22-E_pg23-Q28N*** using the PCR-based SLIM mutagenesis method.

- **pporQ_amp_Q1N** for strain **porQ^Q38N^** - based on plasmid pporQ_amp that introduces the ampicillin cassette downstream of the *porQ* gene. Mutation of the Gln38 to Asp (Q38N) was inserted with primers porQ_Q1N-Fs, porQ_Q1N-Ft, porQ_Q1N-Rs, porQ_Q1N-Rt, using the PCR-based SLIM mutagenesis method.

- **pPorT_his** for strain **porT^his^** - based on plasmid pPorTAtB-C (5) that introduces the tetracycline cassette downstream the *porT* gene. C- terminal His-tag was inserted with Gibson assembly of porT_his_Fr, porT_his_Rv amplified intermediate vector.

- **pPorT_his_Q1N** for strain **porT^Q30N.his^** based on plasmid pporT_his. Mutation of the Gln30 to Asn (Q30N) was inserted with primers porT_Q1N-Fs, porT_Q1N-Ft, porT_Q1N-Rs, porT_Q1N-Rt using the PCR-based SLIM mutagenesis method.

- **pEP2_his** for strain **porG^his^** was commercially synthesized (based on the *CF003_RS11665* gene, *P. gingivalis* optimized codon usage) with C-terminal His-tag.

- **pEP2_his_mut** for strain **porG^Q29N.his^** was commercially synthesized (based on the *CF003_RS11665* gene, *P. gingivalis* optimized codon usage) with mutation of the Gln29 to Asn (Q29N).

**- pEP1_His_pUC57** for strain **PG_0076^his^** was commercially synthesized (based on the *CF003_RS11120* gene, *P. gingivalis* optimized codon usage) with C-terminal His-tag.

**- pEP1_His**_mut for strain **PG_0076^Q25N.his^** was commercially synthesized (based on the *CF003_RS11120* gene, *P. gingivalis* optimized codon usage) with mutation of the Gln25 to Asn (Q25N).

**- pPLUSPg0319&Pg0320-T** master plasmid for PG_0320 mutagenesis, created with the Gibson assembly method and based on vector pUC19. Individual fragments were amplified by suitable primers; pUC19 (2659 bp): wbpB_puc19F and wbpB_puc19R, UP (2297 bp): D319i320UPF and 571P319i320UPR; DW (748 bp): D319i320DWF and D319i320DWR; antibiotic: ORF of tetracycline (2628 bp): wbpBtetF and wbpBtetR.

- **pPLUSPg0320His-T** for strain **PG_0320^his^**, based on plasmid pPLUSPg0319&Pg0320-T, that introduced the tetracycline cassette downstream the PG_0320 gene. Next, C-terminal His-tag was inserted with primers PG0320HisFs, PG0320HisRs, PG0320HisFt, PG0320HisRt using the PCR-based SLIM mutagenesis method.

- **pPLUSPg0320HisQ1N-T,** for strain **PG_0320^Q23N.his^**, based on plasmid pPLUSPg0320His-T, mutation of Gln23 to Asn (Q23N) was inserted with Gibson assembly of the 624_320_Q1N_F, 625_320_Q1N_R amplified intermediate vector.

- **pEP3_his** for strain **PG_1788^his^** was commercially synthesized (based on the *CF003_RS19155* gene, *P. gingivalis* optimized codon usage) with C-terminal His-tag.

- **pEP3_his_mut** for strain **PG_1788^Q21N.his^** was commercially synthesized (based on *CF003_RS19155* gene, *P. gingivalis* optimized codon usage) with mutation of Gln21 to Asn (Q21N).

- **pEP4_his** for strain **PG_0449^his^** was commercially synthesized (based on the *CF003_RS12850* gene, *P. gingivalis* optimized codon usage) with C-terminal His-tag.

- **pEP4_his_mut** for strain **PG_0449^Q22N.his^** was commercially synthesized (based on the *CF003_RS12850* gene, *P. gingivalis* optimized codon usage) with mutation of Gln22 to Asn (Q22N).

***-* pSovAeB** for strain ΔRgpAΔSov, and RgpB^Q25N^ΔRgpAΔSov, obtained earlier (2), with the tetracycline cassette.

- **pPorU/pUC19/Erm** for strain RgpA^Q24N^ΔporU, obtained earlier (6), with the erythromycin cassette.

- **p23AeB** for strain Kgp^Q20N^Δ*porV*, obtained earlier (7), with the erythromycin cassette.

In some experiments expression of the PgQC gene was driven from vectors based on pTIO2-tet (originating from the pTIO-1plasmid (8)) maintained in the *P. gingivalis* cytoplasm. ExpTIO2-tet is a modification of the pTIO2-tet plasmid carrying a RagAB promoter sequence. The PgQC gene and expTIO2-tet were amplified with primers listed in Table S2. Product assembly with In-Fusion® HD Cloning Kit resulted in the expTIO-QC-tet plasmid, which was transformed firstly to the *E. coli* S17-1 strain and subsequently conjugated to *P. gingivalis* W83. Those manipulations generated the QC^m^ strain (the merodiploid strain with both, the plasmid and genomic copy of QC) and the QC^p^ strain (with only plasmid-driven QC expression). In ExpTIO-QC-tet the QC catalytic residue of Asp was mutated to Ala (D126A) using QC- D126A primers and the SLIM method giving yielding the expTIO-QC-tet-D126A plasmid. After its conjugation to *P. gingivalis,* the dominant negative merodiploid PgQC (QC^mD126A^) strain was obtained. The experimental control strain carried an empty pTIO2-tet plasmid.

Table S1. List of strains used in this study.

| **Strains** | | **Description** (*genotype;* resistance) | **Source/**  **reference** |
| --- | --- | --- | --- |
| *Escherichia coli* | | | |
|  | DH5alfa | General cloning host | Thermofisher |
|  | S17-1 | Used for *P. gingivalis* conjugation | (1) |
| *Porphyromonas gingivalis* W83 | | | |
|  | WT | Wild type | ATCC |
|  | ΔPG_2158 | *PG_2158:: ermF*; Em^r^ | This study |
|  | ΔPG_2159 | *PG_2159::ermF*; Em^r^ | This study |
|  | QC^+^ | *PG_2157+ ermF*; Em^r^ | This study |
|  | PG_2159^+^ | *PG_2159+* *ermF*; Em^r^ | This study |
|  | pTIO2-tet | *WT, empty pTIO2-tet* ; Tet^R^ | (2) |
|  | QC^p^ | *PG_2157*::*ermF, expTIO-QC-tet* ; Em^r^, Tet^R^ | This study |
|  | QC^m^ | *WT, expTIO-QC-tet* ; Tet^R^ | This study |
|  | QC^mD126A^ | *WT, expTIO-QC-tet-D126A* ; Tet^R^ | This study |
|  | QC^C20Q^ | *PG_2157 C20Q +ermF; Em^r^* | This study |
|  | PgQC^strep^ | *PG_2157* *333insWSHPQFEK*+ *ermF*; Em^r^ | This study |
|  | PmQC^strep^ | *PG_2157::WP_052080475 (19-333::20-334), 334insWSHPQFEK*+ *ermF*; Em^r^ | This study |
|  | PsQC^strep^ | *PG_2157::KXB76915(19-333::20-330), 330insWSHPQFEK*+ *ermF*; Em^r^ | This study |
|  | TfQC^strep^ | *PG_2157::* *KKY61917.1 (19-333::20-341), 341insWSHPQFEK*+ *ermF*; Em^r^ | This study |
|  | PiQC^strep^ | *PG_2157::* *AFJ09628.1(19-333::20-344), 344insWSHPQFEK*+ *ermF*; Em^r^ | This study |
|  | NsQC^strep^ | *PG_2157::* WP_042277877*(19-333::18-348), 341insWSHPQFEK*+ *ermF*; Em^r^ | This study |
|  | PedgQC^strep^ | *PG_2157::* *WP_057931203 (19-333::18-326), 326insWSHPQFEK*+ *ermF*; Em^r^ | This study |
|  | porV^Q28N^ | *porV Q28N+ ermF*; Em^r^ | This study |
|  | ΔporV | *porV::ermF*; Em^r^ | (7) |
|  | Kgp^Q20N^ΔporV | *porV::ermF; Kgp Q20N+ cepA*; Em^r^, Ap^R^ | This study |
|  | PG_0076^his^ | *PG_0076 313ins6H+ ermF*; Em^r^ | This study |
|  | PG_0076^Q25N.his^ | *PG_0076 Q25N 313ins6H+ ermF*; Em^r^ | This study |
|  | porQ^Q22N^ | *porQ Q22N+ cepA*; Ap^R^ | This study |
|  | porT^his^ | *porT 244ins6H+ tet*; Tet^R^ | This study |
|  | porT^Q30N.his^ | *porT Q30N 244ins6H+ tet*; Tet^R^ | This study |
|  | porG^his^ | *porG 235ins6H+ ermF*; Em^r^ | This study |
|  | porG^Q29N.his^ | *porG Q29N 235ins6H+ ermF*; Em^r^ | This study |
|  | PG_0320^his^ | *PG_0320 362ins6H+ tet*; Tet^R^ | This study |
|  | PG_0320^Q23N.his^ | *PG_0320 Q23N 362ins6H+ tet*; Tet^R^ | This study |
|  | PG_1788^his^ | *PG_1788 262ins6H+ ermF*; Em^r^ | This study |
|  | PG_1788^Q21N.his^ | *PG_1788 Q21N 262ins6H+ ermF*; Em^r^ | This study |
|  | PG_0449^his^ | *PG_0449 460ins6H+ ermF*; Em^r^ | This study |
|  | PG_0449^Q22N.his^ | *PG_0449 Q22N 460ins6H+ ermF*; Em^r^ | This study |
|  | ΔporU | *ΔporU::ermF*; Em^r^ | (6) |
|  | RgpA^Q24N^ΔporU | *ΔporU::ermF, RgpA Q24N+ tet*; Tet^R^; | This study |
|  | ΔSov ΔRgpA | *ΔSov::tet*, *ΔRgpA::cat*; Tet^R^, Cm^r^ | (2) |
|  | RgpB^Q25N^ΔRgpAΔSov | *ΔSov::tet, ΔRgpA::cat,* *RgpB Q25N+ ermF*; Tet^R^, Em^r^, Cm^r^ | This study |

Table S2- List of plasmids used in this study.

| **Plasmids** | **Relevant features** | **Source** |
| --- | --- | --- |
| pURgpB-E | Source of *ermF* cassette | (2) |
| pT-COW | Source of *tetQ* cassette | (1) |
| pQCdel-E | Suicide plasmid for *QC* (*PG_2157)* deletion | This study |
| pQCdelKX-E | Suicide plasmid for *QC (PG_2157)* deletion, modified *pQCdel-E* | This study |
| pUCQC1 | Suicide plasmid for deletion of *PG_2158* | This study |
| pUCQC2-E | Suicide plasmid for polar effect control of *QC* deletion. | This study |
| pUCQC3 | Suicide plasmid for deletion of *PG_2159* | This study |
| pUCQC5 | Suicide plasmid for polar effect control of *PG_2159* deletion | This study |
| pUCQC2-E-D126A | Suicide plasmid for substitution D126A in QC, derivative of *pUCQC2-E* | This study |
| pUCQC2-strep | Suicide plasmid for Strep-tag insertion at C-terminal QC end, derivative of pUCQC2-E | This study |
| pUCQCdelKX-E_pmac-strep | Suicide plasmid for *Porphyromonas macacae QC* insertion in PgQC locus. Derivative of pQCdelKX-E | This study |
| pQCdelKX-E_psom-strep | Suicide plasmid for *Porphyromonas somerae QC* insertion in PgQC locus. Derivative of pQCdelKX-E | This study |
| pQCdelKX-E-nsed-strep | Suicide plasmid for *Nonlabens sediminis QC* insertion in PgQC locus. Derivative of pQCdelKX-E | This study |
| pQCdelKX-E-TF_strep | Suicide plasmid for *Tannerella forsythia QC* insertion in PgQC locus. Derivative of *pQCdelKX-E_psom-strep* | This study |
| pQCdelKX-E_PI-strep | Suicide plasmid for *Prevotella intermedia QC* insertion in PgQC locus. Derivative of *pQCdelKX-E_psom-strep* | This study |
| pQCdelKX-E_pedg-strep | Suicide plasmid for *Pedobacter ginsenosidimutan QC* insertion in PgQC locus. Derivative of pQCdelKX-E | This study |
| pQCdelKX-E_aint-strep | Suicide plasmid for *Alistipes indistinctus QC* insertion in PgQC locus. Derivative of pQCdelKX-E | This study |
| pQCdelKX-E_bint-strep | Suicide plasmid for *Barnesiella intestinihominis* *QC* insertion in PgQC locus. Derivative of pQCdelKX-E | This study |
| pg22-E | Suicide master plasmid for porV mutagenesis | (2) |
| pg22-E_pg23-Q28N | Suicide plasmid for substitution Q28N in *porV*, derivative of pg22-E | This study |
| pNKgp-cep-Q20N | Suicide plasmid for substitution Q20N in Kgp, in ΔporV strain | (9) |
| pEP-his | Suicide plasmid for 6xHis-tag insertion at C-terminal end of PG_0076 | This study |
| pEP_Q24N-his | Suicide plasmid for 6xHis-tag insertion at C-terminal end of PG_0076 and N-terminal Q24N mutagenesis | This study |
| pTIO-1 | *P. gingivalis* expression plasmid, with erythromycin resistance | (8) |
| pTIO2-tet | *P. gingivalis* expression plasmid, with RagAB promoter and with tetracycline resistance, derivative of pTIO-1 plasmid | This study |
| expTIO-QC-tet | *P. gingivalis* expression plasmid, with RagAB promoter for QC overexpression derivative of pTIO2-tet, carrying tetracycline resistance | This study |
| expTIO-QC-tet-D126A | Plasmid for catalytically inactive QC overexpression derivative of *expTIO-QC-tet* | This study |
| pPorQ_amp | Suicide master plasmid for porQ mutagenesis. | (10) |
| pPorQ_amp_Q1N | Suicide plasmid for substitution Q22N in *porQ*, derivative of pPorQ_amp | This study |
| pPorTAtB-C | Suicide master plasmid for porT mutagenesis. | (5) |
| pPorT_his | Suicide plasmid for 6xHis-tag insertion at C-terminal end of porT, derivative of pporTAtB-C | This study |
| pPorT_his_Q1N | Suicide plasmid for substitution Q30N in *porT* and 6xHis-tag insertion at C-terminal end, derivative of pPorT_his | This study |
| pEP2_his | Suicide plasmid for 6xHis-tag insertion at C-terminal end of porG | This study |
| pEP2_his_mut | Suicide plasmid for 6xHis-tag insertion at C-terminal end of porG and N-terminal Q29N mutagenesis | This study |
| pPLUSPg0319&Pg0320-T | Suicide master plasmid for PG_0320 mutagenesis. | This study |
| pPLUSPg0320His-T | Suicide plasmid for 6xHis-tag insertion at C-terminal end of PG_0320 | This study |
| pPLUSPg0320HisQ1N-T | Suicide plasmid for 6xHis-tag insertion at C-terminal end of PG_0320 and N-terminal Q23N mutagenesis | This study |
| pEP3_his | Suicide plasmid for 6xHis-tag insertion at C-terminal end of PG_1788 | This study |
| pEP3_his_mut | Suicide plasmid for 6xHis-tag insertion at C-terminal end of PG_1788 and N-terminal Q21N mutagenesis | This study |
| pEP4_his | Suicide plasmid for 6xHis-tag insertion at C-terminal end of PG_0449 | This study |
| pEP4_his_mut | Suicide plasmid for 6xHis-tag insertion at C-terminal end of PG_0449 and N-terminal Q22N mutagenesis | This study |
| pSovAeB | Suicide plasmid for deletion of *Sov* | (2) |
| pPorU/pUC19/Erm | Suicide plasmid for deletion of *porU* | (6) |
| p23AeB | Suicide plasmid for deletion of *porV* | (7) |

Table S3- List of primers used in this study.

| **Primers** | **Sequence (5’ – 3’)** |
| --- | --- |
| **Plasmids *de novo* construction - genome modification** | |
| **pQCdel-E** | |
| QC_AF_EcoRI | TCTGGAATTCAGAACCCATGCCGGCGG |
| QC_AR_KpnI | GTCTGGTACCATCGTTCTTTCCCTGATAGTGC |
| Erm_F_KpnI | GCGCGGTACCATGACAAAAAAGAAATTGCC |
| Erm_R_XbaI | CGGGTCTAGACTACGAAGGATGAAATTT |
| QC_BF_XbaI | CCGGTCTAGACTATTCACTCATTAGCTTATCC |
| QC_BR_HindIII | GCCAAAGCTTCGACCAGAAGACCGAG |
| **pQCdelKX-E** | |
| QCdelKFs | AAGAAATTGCCCGTTCGTTTTAC |
| QCdelKFt | AAGAACGATATGACAAAAAAGAAATTGCCCGTTCGTTTTAC |
| QCdelKRs | TCCCTGATAGTGCAGGGCAA |
| QCdelKRt | TTTTGTCATATCGTTCTTTCCCTGATAGTGCAGGGCAA |
| QCdelXFs | TATTCACTCATTAGCTTATCCCA |
| QCdelXFt | AATTTCATCCTTCGTAGCTATTCACTCATTAGCTTATCCCA |
| QCdelXRs | TTTCAGGGACAACTTCCAG |
| QCdelXRt | GCTACGAAGGATGAAATTTTTCAGGGACAACTTCCAG |
| **pUCQC1** | |
| QCc1_A-F | GACGGCCAGTGAATTCCCCACCTTTGCCCTGCAC |
| QCc1_A-R | GGGATAAGCTAATGAGTGAATAGTCAGTG |
| QCc1_E-F | TCATTAGCTTATCCCATGACAAAAAAGAAATTGCCCG |
| QCc1_E-R | CAGATGATCCATGAACTACGAAGGATGAAATTTTTCAGGG |
| QCc1_B-F | TTCATGGATCATCTGACCGTCATCATAG |
| QCc1_B-R | CCAAGCTTGCATGCCTGCAGTCAGCCTCTGGCAGAGCGTT |
| **pUCQC2-E** | |
| QCc1_A-F | GACGGCCAGTGAATTCCCCACCTTTGCCCTGCAC |
| QCc1_A-R | GGGATAAGCTAATGAGTGAATAGTCAGTG |
| QCc1_E-F | TCATTAGCTTATCCCATGACAAAAAAGAAATTGCCCG |
| QCc2_E-R | TATACTTGGCATGGACTACGAAGGATGAAATTTTTCAGGG |
| QCc2_B-F | TCCATGCCAAGTATAAACGAAATACAGG |
| QCc2_B-R | CCAAGCTTGCATGCCTGCAGGGCAAAGCGAGTAACCAG |
| **pUCQC3** | |
| QCc1_A-F | GACGGCCAGTGAATTCCCCACCTTTGCCCTGCAC |
| QCc3_A-R | CATGAATCAGGCCTCCCCACCTT |
| QCc3_E-F | GAGGCCTGATTCATGACAAAAAAGAAATTGCCCGTTCG |
| QCc3_E-R | AAGCAAATGTCGTGGCTACGAAGGATGAAATTTTTCAGGG |
| QCc3_B-F | CCACGACATTTGCTTTTCCCTCA |
| QCc3_B-R | CCAAGCTTGCATGCCTGCAGGGCAACAGCCCAAATAAGCC |
| **pUCQC5** | |
| QCc5_A-F | GACGGCCAGTGAATTCCTCGGAGGTATTTATGCAGG |
| QCc5_A-R | GAACGCTCTGCCAGAGGCTGATTC |
| nQCc3_B-F | TAGCCACGACATTTGCTTTTCCCTCA |
| QCc3_B-R | CCAAGCTTGCATGCCTGCAGGGCAACAGCCCAAATAAGCC |
| QCc5_E-F | GCCAGAGGCTGATTCATGACAAAAAAGAAATTGCCCGTTCG |
| QCc3_E-R | AAGCAAATGTCGTGGCTACGAAGGATGAAATTTTTCAGGG |
| ***pUCQCdelKX-E_pmac-strep*** | |
| QC_pUC_Fr | ATGACAAAAAAGAAATTGCCCGT |
| QCdelA-R | ATCGTTCTTTCCCTGATAGTGCAG |
| GQC.sp_Fr | TATCAGGGAAAGAACGATATGAAAAGACTGATAACAACAGGA |
| QC_Pmac_Rv | CAATTTCTTTTTTGTCATCTACTGCGGCTGATATTCATA |
| QC_strepG_Fr | TGGTCTCATCCTCAGTTCGAGAAGTAGATGACAAAAAAGAATTGCCCGT |
| pmac_strepG_Rv | CTTCTCGAACTGAGGATGAGACCACTGCGGCTGATATTCATACAGCA |
| ***pQCdelKX-E_psom-strep*** | |
| QC_pUC_Fr | ATGACAAAAAAGAAATTGCCCGT |
| QCdelA-R | ATCGTTCTTTCCCTGATAGTGCAG |
| GQC.sp_Fr | TATCAGGGAAAGAACGATATGAAAAGACTGATAACAACAGGA |
| QC_Psom_Rv | CAATTTCTTTTTTGTCATCTAGATATCTTCGCGGATCGTCGT |
| QC_strepG_Fr | TGGTCTCATCCTCAGTTCGAGAAGTAGATGACAAAAAAGAATTGCCCGT |
| psom_strepG_Rv | CTTCTCGAACTGAGGATGAGACCAGATATCTTCGCGGATCGTCGT |
| ***pQCdelKX-E-nsed-streptag*** | |
| QC_pUC_Fr | ATGACAAAAAAGAAATTGCCCGT |
| QCdelA-R | ATCGTTCTTTCCCTGATAGTGCAG |
| GQC.sp_Fr | TATCAGGGAAAGAACGATATGAAAAGACTGATAACAACAGGA |
| QC_nsed_Rv | CAATTTCTTTTTTGTCATCTACTGCAGTTCGATTTCGAA |
| QC_strepG_Fr | TGGTCTCATCCTCAGTTCGAGAAGTAGATGACAAAAAAGAATTGCCCGT |
| nsed_strepG_Rv | CTTCTCGAACTGAGGATGAGACCACTGCAGTTCGATTTCGAACAGC |
| ***pQCdelKX-E-TF_streptag*** | |
| QC_TF_Fr | GCTACACTCTCTGCCTGTTGCGGTCAGAAAAATAC |
| QC_TF_Rv | CTACTTCTCGAACTGAGGATGAGACCAATGATTATAAATCACATTCAAGATCG |
| QC_strepG_Fr | TGGTCTCATCCTCAGTTCGAGAAGTAGATGACAAAAAAGAAATTGCCCGT |
| QC_PS_Rv | GGCAGAGAGTGTAGCAGCCAGT |
| ***pQCdelKX-E_PI-strep*** | |
| QC_PI_Fr | GCTACACTCTCTGCCTGCAAAGGAAAATCGTCTAACAA |
| QC_PI_Rv | CTACTTCTCGAACTGAGGATGAGACCACATGCTGTAAAGCACCTGTAT |
| QC_strepG_Fr | TGGTCTCATCCTCAGTTCGAGAAGTAGATGACAAAAAAGAAATTGCCCGT |
| QC_PS_Rv | GGCAGAGAGTGTAGCAGCCAGT |
| ***pQCdelKX-E_pedg-strep*** | |
| QC_pUC_Fr | ATGACAAAAAAGAAATTGCCCGT |
| QCdelA-R | ATCGTTCTTTCCCTGATAGTGCAG |
| GQC.sp_Fr | TATCAGGGAAAGAACGATATGAAAAGACTGATAACAACAGGA |
| QC_pedg_Rv | CAATTTCTTTTTTGTCATCTACTTTTCGCGATAGATCGT |
| QC_strepG_Fr | TGGTCTCATCCTCAGTTCGAGAAGTAGATGACAAAAAAGAATTGCCCGT |
| pedg_strepG_Rv | CTTCTCGAACTGAGGATGAGACCACTTTTCGCGATAGATCGTTTCCA |
| ***pPLUSPg0319&Pg0320-T*** | |
| wbpB_puc19F | GTCGACCTGCAGGCATGCA |
| wbpB_puc19R | GAATTCACTGGCCGTCGTTTTAC |
| D319i320UPF | CGACGGCCAGTGAATTCGGCAGTTCTGTATGGTTGTAA |
| D319i320UPR | GTTAAGGAGATAATTCGTTGTCTACAGTTCGGCTTTGACCT |
| D319i320DWF | AGAAGCATTAGAACTTGGCCTTTTCCCCGTAGAGCTGCT |
| D319i320DWR | CATGCCTGCAGGTCGACCGCGCTTGATACCCTGAAGT |
| wbpBtetF | ACAACGAATTATCTCCTTAACG |
| wbpBtetR | GCCAAGTTCTAATGCTTCTATC |
| **Plasmids *de novo* construction- cytoplasmic expression** | |
| ***expTIO-QC-tet*** | |
| expTIO_QCfor | TGCCAAGTTCTAATGCTTCTATCT |
| expTIO_QCrev | AGACTTTTCTTTTGCGTTAAACTT |
| insQCfor | GCAAAAGAAAAGTCTATGAAAAGACTGATAACAACAGGAG |
| insQCrev | CATTAGAACTTGGCATCAGTGTGAAGCGGCTTTC |
| ***Plasmids’ mutagenesis*** | |
| ***QC- D126A*** | |
| QCdelDAFs | GTCTGCGATCAGGATGCCA |
| QCdelDAFt | CACTGGGCCACGCGGCCGGTCTGCGATCAGGATGCCA |
| QCdelDARs | AGCCATCAGTAGCATACGCCC |
| QCdelDARs | CGGCCGCGTGGCCCAGTGAGCCATCAGTAGCATACGCCC |
| ***QC- Strep-tag*** | |
| QCstreptagR | CTTCTCGAACTGAGGATGAGACCAGTGTGAAGCGGCTTTCACCTG |
| QCstreptagF | TGGTCTCATCCTCAGTTCGAGAAGTGACTATTCACTCATTAGCTTATCCC |
| ***pg22-E_pg23-Q28N*** | |
| Pg23-Q1N_Fs | AATGTGGTACACACCTCTGTGC |
| Pg23-Q1N_Ft | AAGGCTAACGAGCAACTGAATGTGGTACACACCTCTGTGC |
| Pg23-Q1N_Rs | CATAGCCTTTGGAGCAAAAAGGAAC |
| Pg23-Q1N_Rt | CAGTTGCTCGTTAGCCTTCATAGCCTTTGGAGCAAAAAGGAAC |
| ***pPorQ_amp_Q1N*** | |
| porQ_Q1N-Fs | GGTGTTTCATTTTCTGAACCT |
| porQ_Q1N-Ft | GGTGCTAACCAAGAGAAGCAGGTGTTTCATTTTCTGAAC |
| porQ_Q1N-Rs | TGCCGAAAACACAAGAGAAAG |
| porQ_Q1N-Rt | TGCTTCTCTTGGTTAGCACCTGCCGAAAACACAAGAGAAAG |
| ***pPorT_his*** | |
| porT_his_Fr | CACCATCACCATCACCATTAGTTGTCACGCTCTTTTCGACA |
| porT_his_Rv | ATGGTGATGGTGATGGTGCTCGAAATTGAACGTAAGCAT |
| ***pPorT_his_Q1N*** | |
| porT_Q1N-Fs | GTGTGATAACATCGGGAA |
| porT_Q1N-Ft | AAAGATAACGACGCTCGTGTGTGATAACATCGGGAA |
| porT_Q1N-Rs | TGGATGATTATAAGTTGATCTA |
| porT_Q1N-Rt | ACGAGCGTCGTTATCTTTTGGATGATTATAAGTTGATCTA |
| ***pPLUSPg0320His-T*** | |
| PG0320HisFs | TAGACAACGAATTATCTCCTTAACG |
| PG0320HisRs | CAGTTCGGCTTTGACCTTGATA |
| PG0320HisFt | CATCACCATCACCATCACTAGACAACGAATTATCTCCTTA |
| PG0320HisRt | GTGATGGTGATGGTGATGCAGTTCGGCTTTGACCTTGATA |
| ***pPLUSPg0320HisQ1N-T*** | |
| 624_320_Q1N_F | GCTAACGAGAGTCCGGAAATACAGGTCGAGGAGCAG |
| 625_320_Q1N_R | CCGGACTCTCGTTAGCTGTCAGCAACAGAGGAGTCA |
| **Real time PCR primers** | |
| RpoB_F | GGAAGAGAAGACCGTAGCACAAGG |
| RpoB_R | GAGTAGGCGAAACGTCCATCAGGT |
| 23qPCR_F | TGCGGAGAGCTTGTGGTCGT |
| 23qPCR_R | ACAACAGCCCCGTCCCGAGA |
| KgpqPCR_F | GTCCCTTTTGGGAGTTGGTCT |
| KgpqPCR_R | CGGAATTGACACTGAGGCGA |
| qPCR_Pg0076_F | TCCCGATTTCTGCTCCAACC |
| qPCR_pg0076_R | CAGATTGGCATAGCCCCGAT |
| porG_qPCR_F | TTTGGAGTTGGTCGGACGTT |
| porG_qPCR_R | CGCTTCTAACGTGGAGCTGA |
| Pg1779_qPCR_F | GACTTGGCCGAAATGCACTC |
| Pg1779_qPCR_R | ACATCGTAGAAGGAACCGCC |
| Pg0449_qPCR_F | CTTCCGACTGCGAGACACAT |
| Pg0449_qPCR_R | CCTCTTTGGCAAGGCCCATA |
| RgpB_qRTFor | AGCGTACTTTCGGAGGTGTC |
| RgpB_qRTRev | ATCACCATCGTCAGAGAGCG |
| 677qPCR_F | ATCGCCCCTACGCCGACTACA |
| 677qPCR_R | ACGGAGAAAGAGGCCACGGGT |
| 543qPCR_F | GCTCGGCTTTTCCCGCAGTT |
| 543qPCR_R | TGTTGCCGCCTTCCACCTCG |
| HRgpA For qPCR1 | ACGCTTCCCATTCTATCACG |
| HRgpA Rev qPCR2 | CTCCCGGGAAGAATTTGTTT |
| qPCR_Pg320f | CCGTACCTCGCAATATGCCT |
| qPCR_Pg320r | GCAAGGAGACCATGACGGAA |

**References**

1. Bélanger M, Rodrigues P, Progulske-Fox A. 2007. Genetic manipulation of Porphyromonas gingivalis. Curr Protoc Microbiol Chapter 13:Unit13C.2.

2. Mizgalska D, Goulas T, Rodríguez-Banqueri A, Veillard F, Madej M, Małecka E, Szczesniak K, Ksiazek M, Widziołek M, Guevara T, Eckhard U, Solà M, Potempa J, Gomis-Rüth FX. 2021. Intermolecular latency regulates the essential C-terminal signal peptidase and sortase of the Porphyromonas gingivalis type-IX secretion system. Proc Natl Acad Sci U S A 118:e2103573118.

3. Chiu J, Tillett D, Dawes IW, March PE. 2008. Site-directed, Ligase-Independent Mutagenesis (SLIM) for highly efficient mutagenesis of plasmids greater than 8kb. J Microbiol Methods 73:195–198.

4. Gibson DG, Young L, Chuang R-Y, Venter JC, Hutchison CA, Smith HO. 2009. Enzymatic assembly of DNA molecules up to several hundred kilobases. Nat Methods 6:343–345.

5. Nguyen K-A, Żylicz J, Szczesny P, Sroka A, Hunter N, Potempa J. 2009. Verification of a topology model of PorT as an integral outer-membrane protein in Porphyromonas gingivalis. Microbiology (Reading) 155:328–337.

6. Lasica AM, Goulas T, Mizgalska D, Zhou X, de Diego I, Ksiazek M, Madej M, Guo Y, Guevara T, Nowak M, Potempa B, Goel A, Sztukowska M, Prabhakar AT, Bzowska M, Widziolek M, Thøgersen IB, Enghild JJ, Simonian M, Kulczyk AW, Nguyen K-A, Potempa J, Gomis-Rüth FX. 2016. Structural and functional probing of PorZ, an essential bacterial surface component of the type-IX secretion system of human oral-microbiomic Porphyromonas gingivalis. Sci Rep 6:37708.

7. Madej M, Nowakowska Z, Ksiazek M, Lasica AM, Mizgalska D, Nowak M, Jacula A, Bzowska M, Scavenius C, Enghild JJ, Aduse-Opoku J, Curtis MA, Gomis-Rüth FX, Potempa J. 2021. PorZ, an Essential Component of the Type IX Secretion System of Porphyromonas gingivalis, Delivers Anionic Lipopolysaccharide to the PorU Sortase for Transpeptidase Processing of T9SS Cargo Proteins. mBio 12:e02262-20.

8. Tagawa J, Inoue T, Naito M, Sato K, Kuwahara T, Nakayama M, Nakayama K, Yamashiro T, Ohara N. 2014. Development of a novel plasmid vector pTIO-1 adapted for electrotransformation of Porphyromonas gingivalis. J Microbiol Methods 105:174–179.

9. Bochtler M, Mizgalska D, Veillard F, Nowak ML, Houston J, Veith P, Reynolds EC, Potempa J. 2018. The Bacteroidetes Q-Rule: Pyroglutamate in Signal Peptidase I Substrates. Front Microbiol 9:230.

10. Nowakowska Z, Madej M, Grad S, Wang T, Hackett M, Miller DP, Lamont RJ, Potempa J. 2021. Phosphorylation of major Porphyromonas gingivalis virulence factors is crucial for their processing and secretion. Mol Oral Microbiol 36:316–326.
