# Supplementary material for: The Bacteroidetes Q-rule and glutaminyl cyclase activity increase the stability of extracytoplasmic proteins
Source: mBio. 2023 Sep 26;14(5):e00980-23. doi: 10.1128/mbio.00980-23 (PMC10653852; doi:10.1128/mbio.00980-23)
Supplement: Fig. S1 to Fig. S5 — Figures with additional strains phenotypic characterization, and protein sequence alignments. [file mbio.00980-23-s0002.docx]

1. ***Supplementary figures***

**Figure S1.** Gingipain activity assays. (**A** and **B**) Wild-type (WT), ΔPG_2158, ΔPG_2159 and QC^+^ strains. (**C** and **D**) The pTIO2-tet, QC^p^ and QC^m^ strains. Bacterial cultures were harvested at the early stationary phase and gingipain amidolytic activity was determined in whole culture (WC) and cell-free medium for RgpA/B (**A, C**) and Kgp (**B, D**). Data are means ± SD (*n* = 3 technical replicates) and are representative of three independent experiments. Statistical significance was calculated by one-way ANOVA and *post hoc* Bonferroni correction (**p* < 0.05, ***p* < 0.001, ****p* < 0.001, *****p* < 0.0001).

**Figure S2.** Phenotypic comparison of mutant strains expressing PgQC in which the lipobox Cys is replaced with Gln (QC^C20Q^) vs the parental wild-type (WT) strain. (**A**) Growth curves of the *P. gingivalis* WT and QC^C20Q^ strains plotted by monitoring cell density (OD_600_) in liquid cultures for 16 h in triplicate. The pigmentation phenotype shown in the inset was photographed after anaerobic growth for 10 days on blood eTSB agar. (**B**) RgpA/B and (**C**) Kgp activity in whole cultures (WC) at the early stationary phase and in the cell-free medium. Data are means ± SD (*n* = 3 technical replicates) and are representative of three independent experiments. . Statistical significance was calculated using Student’s *t*-test or one-way ANOVA with Tukey’s correction (**p* < 0.05, ***p* < 0.01, *****p* < 0.0001). (**D**) Comparison of QC activity in the control (WT) and QC^C20Q^ strains using chromogenic substrate H-Gln-AMC (0.25 mM). Data are means ± SD (*n* = 3 technical replicates) and are representative of three independent experiments. (**E**) Representative western blots showing the abundance of QC protein in different cell fractions of the WT and QC^20Q^ strains. Fraction designations: WC – whole culture, M – medium, WCE – whole cell extract, PP/CP – periplasm/cytoplasm, CM – cell membranes, IM – inner membranes, OM – outer membranes. Western blots were probed with anti-QC primary antibodies (1) and HRP-conjugated anti-rabbit secondary antibodies.

**Figure S3.** Sequence alignments and gingipain activity assays. Alignment of type II QCs (**A**) (*P. gingivalis* PgQC, *P. macacae* PmQC, *P. somerae* PsQC, *P.* *ginsenosidimutan* PedgQC, *P. intermedia* PiQC, *T. forsythia* TfQC, *B. intestinihominis* BiQC and *Homo sapiens* HsQC and type I QCs (**B**) (*A.* *indistinctus* AiQC, *N. sediminis* NsQC and *Carica papaya* CpQC performed by T-Coffee (https://tcoffee.crg.eu) (2), and visualized by Jalwiev (http://www.jalview.org) (3) (residues are shadowed according to identity score). The red box mark lipidated residues identified by SignalP 6.0 (https://services.healthtech.dtu.dk/services/SignalP-6.0/) (4), and star indicate position of conservative Asp126. (**C**, **D**) Comparison of gingipain activity in *P. gingivalis* mutant strains expressing heterologous QCs. (**C**) RgpA/B and (**D**) Kgp activity was determined in whole cultures (WC) at the early stationary phase and in cell-free medium. Data are means ± SD (*n* = 3 technical replicates) and are representative of three independent experiments. Statistical significance was calculated using Student’s *t*-test or one-way ANOVA with Tukey’s correction (**p* < 0.05, ***p* < 0.01, ****p* < 0.001, *****p* < 0.0001).

**Figure S4.** Pigmentation phenotype and growth curves of different *P. gingivalis* strains. (**A**-**K)** Representative growth curves of the indicated *P. gingivalis* strains plotted by monitoring cell density (OD_600_) in liquid cultures for 16 h performed in triplicate. The insets illustrate the pigmentation phenotype on blood eTSB agar after growth under anaerobic conditions for 10 days.

**Figure S5.** Comparison of gingipain activity in different *P. gingivalis* strains. (**A-H**) RgpA/B (**left panels**) and Kgp (**right panels**) activity in the indicated strains was determined in whole cultures (WC) at the early stationary phase and in cell-free medium. Data are means ± SD (*n* = 3 technical replicates) and are representative of three independent experiments. The statistical significance of differences between the mutated strains and their direct control strains were calculated using Student’s *t*-test or one-way ANOVA with Tukey’s correction (**p* < 0.05, ***p* < 0.01, ****p* < 0.001, *****p* < 0.0001).

**References**

1. Bochtler M, Mizgalska D, Veillard F, Nowak ML, Houston J, Veith P, Reynolds EC, Potempa J. 2018. The Bacteroidetes Q-Rule: Pyroglutamate in Signal Peptidase I Substrates. Front Microbiol 9:230.

2. Notredame C, Higgins DG, Heringa J. 2000. T-coffee: a novel method for fast and accurate multiple sequence alignment 1 1Edited by J. Thornton. Journal of Molecular Biology 302:205–217.

3. Waterhouse AM, Procter JB, Martin DMA, Clamp M, Barton GJ. 2009. Jalview Version 2—a multiple sequence alignment editor and analysis workbench. Bioinformatics 25:1189–1191.

4. Teufel F, Almagro Armenteros JJ, Johansen AR, Gíslason MH, Pihl SI, Tsirigos KD, Winther O, Brunak S, von Heijne G, Nielsen H. 2022. SignalP 6.0 predicts all five types of signal peptides using protein language models. Nat Biotechnol 40:1023–1025.
